# Optimising the identification of causal variants across varying genetic architectures in crops

**DOI:** 10.1111/pbi.13023

**Published:** 2018-11-09

**Authors:** Chenyong Miao, Jinliang Yang, James C. Schnable

**Affiliations:** ^1^ Department of Agronomy and Horticulture University of Nebraska‐Lincoln Lincoln NE USA; ^2^ Center for Plant Science Innovation University of Nebraska‐Lincoln Lincoln NE USA

**Keywords:** genome‐wide association study, polygenic traits, genetic architecture, quantitative genetics

## Abstract

Association studies use statistical links between genetic markers and the phenotype variation across many individuals to identify genes controlling variation in the target phenotype. However, this approach, particularly conducted on a genome‐wide scale (GWAS), has limited power to identify the genes responsible for variation in traits controlled by complex genetic architectures. In this study, we employ real‐world genotype datasets from four crop species with distinct minor allele frequency distributions, population structures and linkage disequilibrium patterns. We demonstrate that different GWAS statistical approaches provide favourable trade‐offs between power and accuracy for traits controlled by different types of genetic architectures. FarmCPU provides the most favourable outcomes for moderately complex traits while a Bayesian approach adopted from genomic prediction provides the most favourable outcomes for extremely complex traits. We assert that by estimating the complexity of genetic architectures for target traits and selecting an appropriate statistical approach for the degree of complexity detected, researchers can substantially improve the ability to dissect the genetic factors controlling complex traits such as flowering time, plant height and yield component.

## Introduction

Association studies in natural populations have been widely adopted as a complement to classical gene mapping and gene knockout approaches in identifying and characterising the functions of specific genes. Association studies identify functionally variable alleles segregating in target species and these alleles can guide breeding efforts in crop and livestock species, as well as provide increasingly accurate predictions of disease risk factors in humans. Advances in genotyping technology have dramatically reduced the barriers to conducting association studies with genome‐wide genetic marker datasets across natural populations. Since becoming feasible in the mid‐2000s, genome‐wide association studies (GWAS) have been successfully used to identify thousands of single nucleotide polymorphisms (SNPs) associated with diseases in human (Burton *et al*., 2007) and complex agricultural traits in plants (Chen *et al*., 2016; Jia *et al*., 2013; Lasky *et al*., 2015; Romero Navarro *et al*., 2017). For most traits analysed, loci identified by GWAS can generally explain only a subset of total genetically controlled phenotypic variation for most traits analysed (Maher, 2008; Manolio *et al*., 2009; Visscher *et al*., 2010). Many explanations have been proposed for this “missing heritability” including epigenetic effects (Gerasimova *et al*., 2013), epistasis (Moellers *et al*., 2017; Visscher *et al*., 2008; Zhang *et al*., 2015), structural variants which are not detected by conventional SNP genotyping (McCarroll, 2008), rare alleles with large effects and common alleles with small effect sizes (Jakobsdottir *et al*., 2009; Pritchard, 2001). While the first two proposed explanations for missing heritability are more difficult to address, both rare alleles with large effect sizes and common alleles with small effect sizes can potentially be identified through increases in the statistical power of GWAS to identify causal variants.

Many traits of interest to biologists are controlled by complex genetic architectures (Huang *et al*., 2012; Lasky *et al*., 2015; Romero Navarro *et al*., 2017) where hundreds, thousands, or the majority of all genes (Boyle *et al*., 2017) may control variation in the target trait. The most straightforward approach to increase the proportion of causal variants identified is to increase the size of genotyped and phenotyped populations. However, increases in population size are expensive and subject to diminishing returns in terms of the improvement of power to detect both rare alleles and alleles with small effect sizes. Improved statistical approaches to isolating a larger proportion of total causal variants controlling complex traits are therefore highly desirable.

Currently, GWAS approaches based on mixed linear models (MLM) are widely employed in both plant and animal systems. MLM‐based approaches are able to control for confounding effects of both population structure and unequal relatedness among individuals, which are left uncontrolled in approaches based on generalised linear models (GLM), at the expense of greater run times. A wide range of different algorithms have been proposed and developed to improve the computational efficiency of MLM, including EMMAX (Kang *et al*., 2010), Compressed‐MLM (Zhang *et al*., 2010), FaST‐LMM (Lippert *et al*., 2011) and GEMMA‐MLM (Zhou and Stephens, 2012). However, because MLM‐based methods are ultimately evaluating the relationship between each genetic marker and the overall variation in a given trait across a population independently, the statistical power of these methods rapidly decreases as the total number of variants controlling variation in a given trait increases, and the proportion of total genetic variance explained by any one locus decreases.

Multi‐locus mixed‐models (MLMM) explicitly identify and control for the effects of large effect loci as fixed effects as these loci are identified by the model. Compared to GLM or MLM which only conduct tests on one marker at a time, the MLMM can test multiple markers simultaneously by fitting the supposed causal variants in the process called “forward‐backward stepwise linear mixed‐model regression” (Segura *et al*., 2012). This approach increases the proportion of the remaining genetic variance explained by the remaining unidentified variants, and increases the statistical power of the method to detect a greater number of causal variants for complex traits. While the high computational cost of MLMM initially acted as a barrier to widespread adoption, a modified method, fixed and random model circulating probability unification (FarmCPU), has dramatically reduced the computational complexity and computing time of this approach (Liu *et al*., 2016). Ongoing optimisation and parallelisation efforts have continued to decrease real‐world run times for MLMM‐based approaches (Schnable and Kusmec, 2017).

A second potential approach to accurately identifying causal variants for traits controlled by complex genetic architectures is the use of Bayesian multiple‐regression methods (Fernando and Garrick, 2013; Fernando *et al*., 2017). The Bayesian‐based approaches fit all the available markers simultaneously, which makes them especially suitable to study highly polygenic traits. Although Bayesian approaches such as BayesA, BayesB, BayesC and BayesCπ have been widely employed in genomic prediction and selection areas (Bernardo and Yu, 2007; Hayes *et al*., 2001; Piepho, 2009; Sun *et al*., 2011; Verbyla *et al*., 2009), they are seldom applied in GWAS, especially in plant GWAS. Several studies have employed Bayesian‐based approaches to identify putative causal variants in animals (Fan *et al*., 2011; Peters *et al*., 2012); however, the performance of these Bayesian methods when employed in GWAS have not been extensively evaluated relative to current non‐Bayesian approaches.

Here, we systematically compared the performance of MLM, FarmCPU and Bayesian‐based (BayesCπ) GWAS approaches across simulated trait datasets containing 2 to 1024 causal variants and different levels of heritability ranging from 0.1 to 1. To capture realistic patterns of minor allele frequency distributions, population structure and linkage disequilibrium, we employed real‐world genotype datasets from four widely studied crop species: rice (*Oryza sativa*), foxtail millet (*Setaria italica*), sorghum (*Sorghum bicolor*) and maize (*Zea mays*) (Jia *et al*., 2013; Lasky *et al*., 2015; McCouch *et al*., 2016; Romay *et al*., 2013). We demonstrate that the power and accuracy of both FarmCPU and BayesCπ to identify causal variants for complex traits exceed conventional MLM‐based approaches. Of the three methods, FarmCPU generally provides the most favourable trade‐off between power and low false discovery rates (FDR) for moderately complex traits controlled by several dozen variants, while the BayesCπ approach provides a more favourable trade‐off for traits controlled by hundreds of more variants. However, the number of casual variants where the cross‐over between the comparative advantages of these two methods occurred varied across species. The results presented here, including a set of 4000 simulated phenotypic datasets generated from four real‐world genotype datasets, will provide both a resource for evaluating future innovations in GWAS software, and information to help researchers select the most effective experimental design and statistical approach for their particular research projects.

## Results

### Characteristics of the four association populations employed in this study

**Figure 1:**
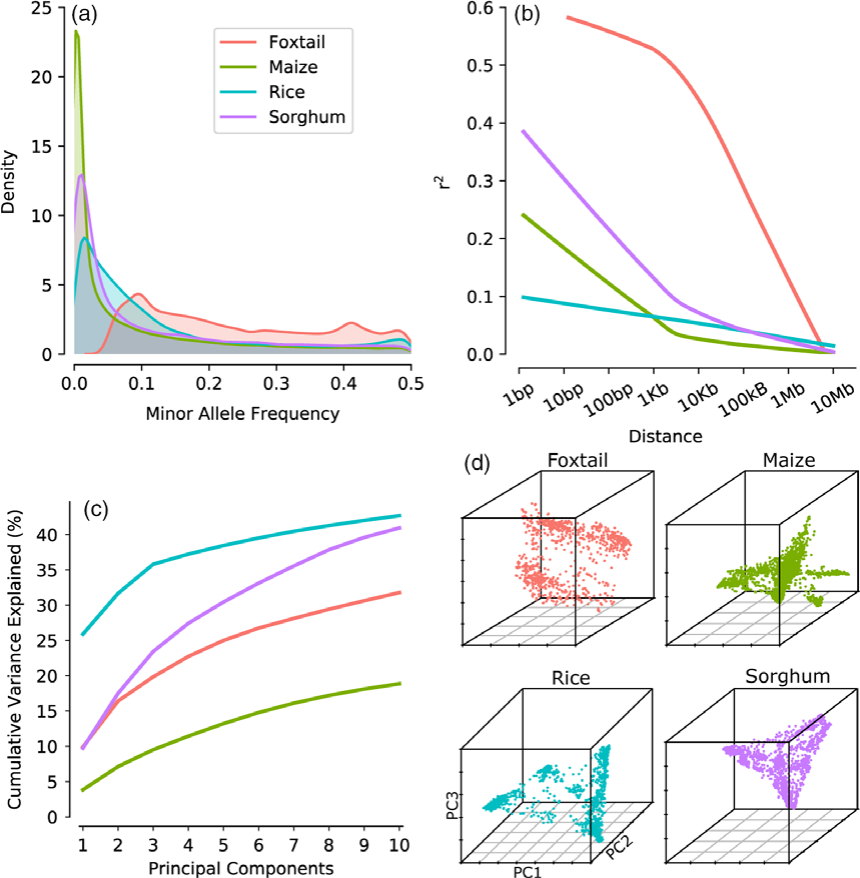
Characterisation of the four association populations and associated genotype datasets employed in this study. (a) Distribution of minor allele frequencies across all genotyped markers in each population. (b) Patterns of linkage disequilibrium decay in each population based on average pairwise *r* ^2^ between genetic markers (Methods). (c) Cumulative proportion of total genotypic variance explained up to ten principal components in each population. (d) PCs distribution for individuals in each population.

Each of the four populations employed in this study presents a different combination of linkage disequilibrium, minor allele frequency distribution and population structure (Figure 1, Table 1). These differences may result from differences in population demographics, criteria used to assemble the populations and genotyping technologies employed in each of the genotype datasets. For example, the comparatively low frequency of rare alleles in rice results from selection loci with more frequent minor alleles prior to microarray design (McCouch *et al*., 2016), while the low frequency of rare alleles in foxtail millet results from a post‐genotyping, prepublication filter for loci with relatively more common minor alleles (Jia *et al*., 2013). Marker selection for inclusion on the rice genotyping array incorporated an explicit counter‐selection against markers in high LD with each other within the resequencing population and the extremely low LD observed in this set of markers is consistent with the LD analysis in the original release paper (McCouch *et al*., 2016). Foxtail millet population exhibited the slowest LD decay, with the average correlation coefficient (*r* ^2^) between genetic markers dropping to 0.25 around 100 kb, consistent with the original description of this dataset (Jia *et al*., 2013). The LD decay curve shown here for maize is somewhat more rapid than was reported in (Romay *et al*., 2013), however, this divergence is likely explained by the Romay *et al*. curve being calculated using a subset of ~22 000 SNPs with low missing data and high minor allele frequencies. With the exception of rice, the patterns of LD decay observed across populations of the remaining three species exhibit a negative correlation with reported outcrossing frequencies for each species (Figure S1). This negative correlation suggests that the difference is the result of biological variation rather than genotyping strategy (Barnaud *et al*., 2008; Djè *et al*., 2004; Gutierrez and Sprague, 1959; Hufford *et al*., 2011; Wang *et al*., 2010).

**Table 1:** Statistical summary of each genotype dataset

Species	Genotyping technology	Genome size (Mb)	LD Decay (Kb)	No. of Accessions	No. of Markers
*Sorghum bicolor*	GBS	732	2	2327	354 940
*Setaria italica*	Low coverage WGS	406	794	916	663 985
*Oryza sativa*	Microarray	372	0.004	1568	629 019
*Zea mays*	GBS	2300	0.063	2503	560 515

### Evaluation of conventional MLM‐based GWAS

**Figure 2:**
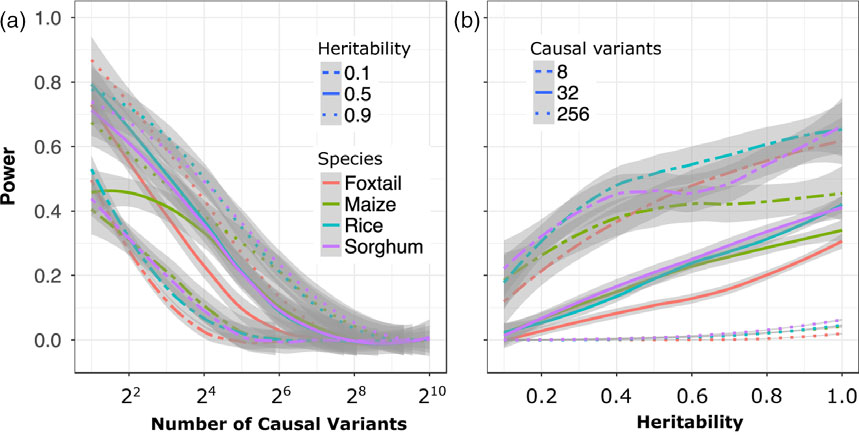
Changes in the power of conventional (MLM‐based) GWAS to identify causal variants in response to changes in heritability and the complexity of the genetic architecture controlling the target trait. Data shown are from foxtail millet. (a) Change in power to detect true positives as the number of causal variants increases under high (0.9), medium (0.5) and low (0.1) levels of heritability. (b) Change in power to detect true positives as heritability decreases for traits controlled by simple (*N* = 8), moderately complex (*N* = 32) and complex (*N* = 256) genetic architectures. Positive calls were defined as those above a Bonferroni corrected *P*‐value cut‐off of 0.05. Comprehensive results from all four populations are available in Figures S2 and S3.

**Figure 3:**
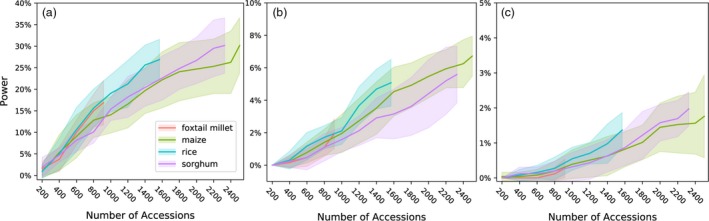
Changes in the power of conventional MLM‐based GWAS to identify causal variants for complex traits in response to increases in population size in each of the four association populations evaluated. (a) a moderately complex trait controlled by 32 loci; (b) a complex trait controlled by 128; (c) a complex trait controlled by 256 loci. All analyses used data from traits with heritability of 0.7. Positive calls were defined as those above a Bonferroni corrected *P*‐value cut‐off of 0.05.

A total of 1000 phenotype datasets were generated per species with ten independent replicates for each possible combination of ten different sets of causal variants and ten different levels of heritability, which represents different levels of genetic architecture complexity. A causal variant was considered to be identified if either the causal SNP selected by the simulations, or one or more markers linked (*r* ^2^ > 0.6) with the causal SNP were identified by a given GWAS analysis. As expected, the power to detect true positives decreased in response to both increases in the number of simulated causal variants controlling the trait and decreases in simulated heritability (Figure 2). The MLM‐based approach failed to identify the vast majority of causal variants for traits controlled by 256 or more loci under whatever levels of heritability (Figures S2 and S3). Consistent with previous theory and studies that both rare alleles and alleles with smaller effect sizes were the least likely to be identified in the MLM‐based GWAS analysis (Figure S4; Table S1). Subsampling of each population was used to evaluate how rapidly the proportion of total causal variants identified increases with increased population size. The effect of increasing population size was relatively more pronounced when genetic architecture was less complex, and smaller increases were observed with increasing population size for more complex genetic architectures (Figure 3).

### Alternative GWAS methods for complex traits

**Figure 4:**
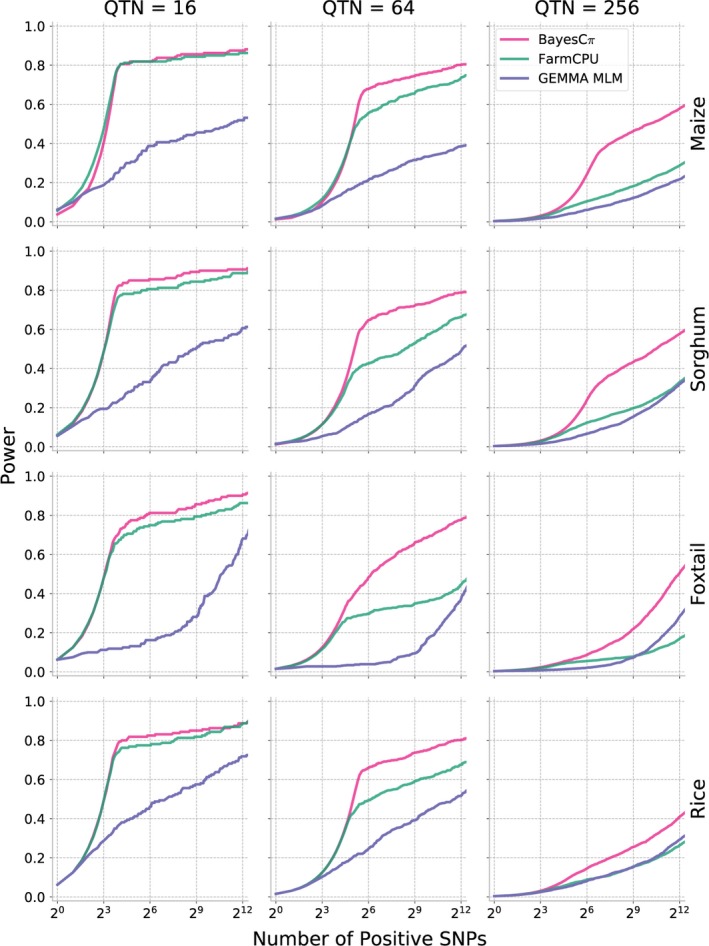
Changes in the power of three GWAS approaches across all four association populations in response to the changes of the statistical threshold employed. To enable comparisons across different methods with different approaches to reporting statistical significance, the *x*‐axis is ordered by the total number of positive genetic markers accepted at a given statistical threshold. Data shown are for traits with increasingly complex genetic architectures with near‐best‐case assumptions for trait heritability (0.9). Results for all other simulated genetic architectures are provided in Figures S5, S6, S9 and S10.

**Figure 5:**
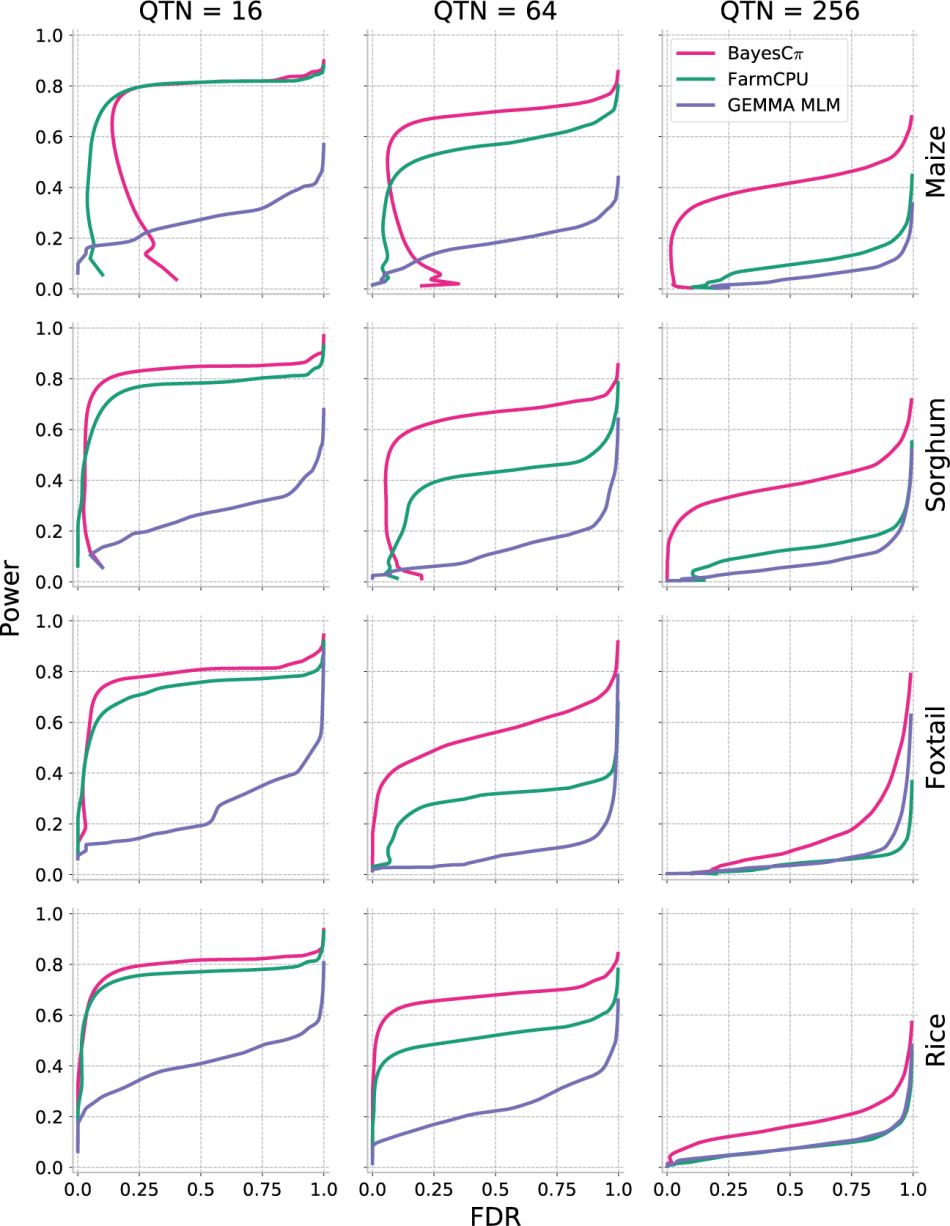
Relationship between power and false discovery rate (FDR) using each GWAS method to analyse simple, medium and complex traits in each population. Data shown are for traits with increasingly complex genetic architectures with near‐best‐case assumptions for trait heritability (0.9). Results for all other simulated genetic architectures are provided in Figures S7, S8, S11 and S12.

As shown above, MLM‐based GWAS identifies only a small proportion of causal variants for complex traits controlled by over hundreds of distinct genetic loci. We next evaluated two methods potentially developed to analyse polygenic traits: FarmCPU (Liu *et al*., 2016) and BayesCπ (Habier *et al*., 2011). To avoid confounding effects from different approaches to scoring the strength of associations between genetic markers and trait variation, cross‐method comparisons are made based on selecting equivalent numbers of positive causal variants in each analysis. The proportion of causal variants detected declines in each species as heritability decreases and as the total number of causal variants controlling the trait increases. However, FarmCPU and BayesCπ both consistently outperformed MLM‐based analysis in terms of both overall proportion of causal variants identified and FDR control (Figures 4 and 5). For moderately complex traits (32, 64 causal variants), the statistical power of BayesCπ and FarmCPU provided approximately equivalent statistical power, however, FarmCPU tends to provide lower false discovery rates than BayesCπ for these genetic architectures (Figures 4 and 5, S5 and S7). For complex traits with high heritability (128, 256 causal variants with *h* ^2^ = 0.9), the BayesCπ approach outperforms FarmCPU on both power and false discovery rate metrics (Figures 4 and 5, S6, and S8). However, we also observed that this advantage is less apparent for traits with medium heritability (*h* ^2^ = 0.7; Figures S10 and S12). Furthermore, once heritability decreased to 0.5, the difference between these two methods was only apparent in the foxtail millet dataset (Figures S9, and S11). The two approaches exhibited similar power to control type I error for traits controlled by simple genetic architectures while BayesCπ exhibited better performance on false‐positive control for traits controlled by moderately and extremely complex genetic architectures (Figures S13 and S14).

A second analysis was conducted utilising published flowering time data for 1371 maize inbred lines from the maize association population utilised in the simulation study above (Romay *et al*., 2013). Flowering time in maize is an extremely complex trait controlled by hundreds of genes and most individual loci explain only extremely small proportions of total phenotypic variance (Buckler *et al*., 2009; Romero Navarro *et al*., 2017). MLM, FarmCPU and BayesCπ analyses identified 12, 20 and 32 markers within this dataset, respectively. To assess accuracy, we employed a set of candidate flowering time genes identified in an independent study utilising distinct genotypic and phenotypic data collected from 4471 maize lines across 22 environments (Romero Navarro *et al*., 2017). Of the signals identified by each algorithm above, 3 (25%), 5 (25%) and 13 (41%) of the markers identified via MLM, FarmCPU and BayesCπ, respectively, were located within 50KB of a flowering time candidate gene identified in the independent and more highly powered study (Appendix S1). Markers associated with three candidate flowering time genes were identified by both FarmCPU and BayesCπ. No overlap of identified candidate genes was observed between candidates identified by MLM and the other two methods.

**Figure 6:**
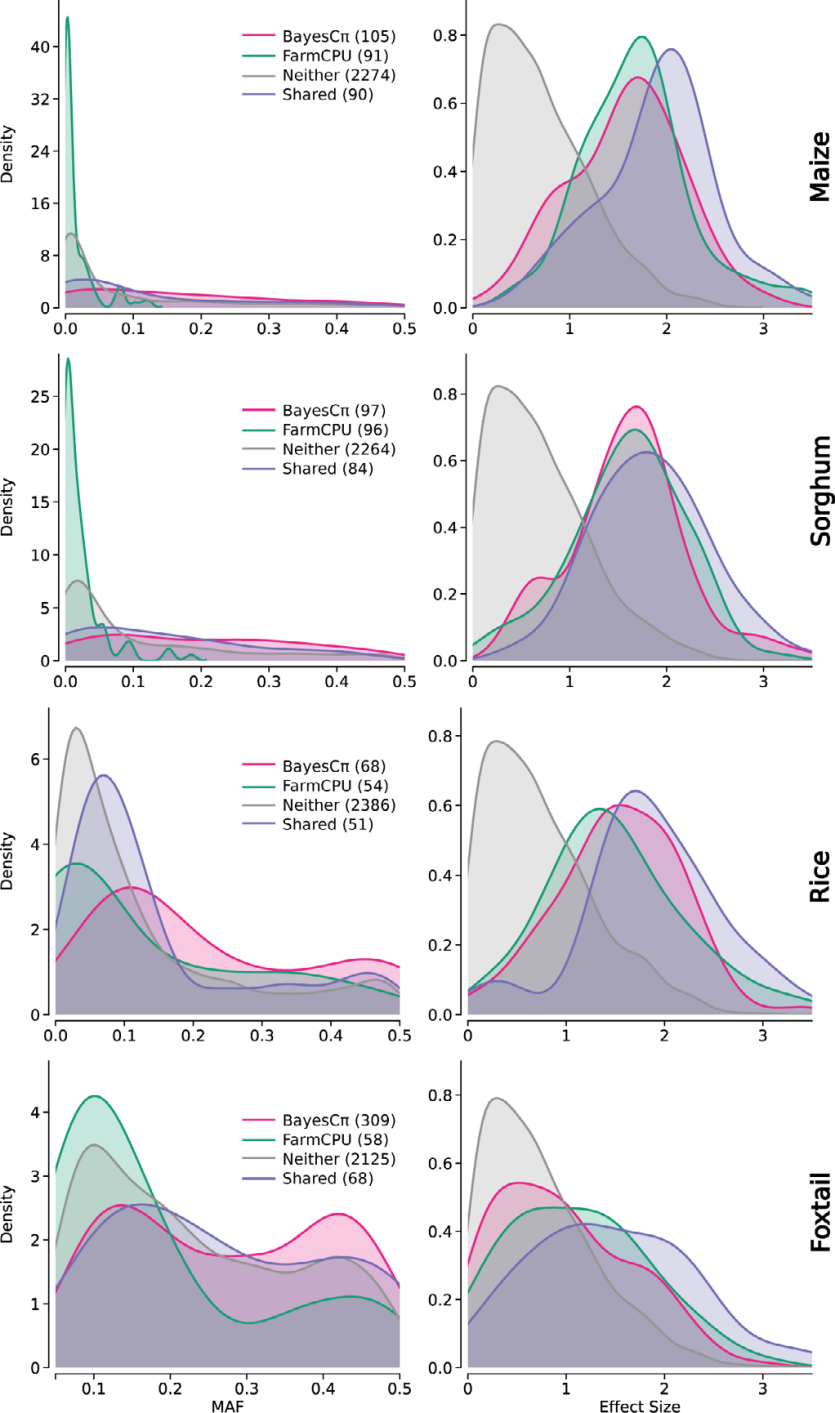
Differences in the characteristics of causal SNPs identified by BayesCπ and FarmCPU in all four species. Distribution of MAF (Left) and absolute effect size (Right) for causal variants identified by both BayesCπ and FarmCPU, only BayesCπ, only FarmCPU, or neither approach. The number of causal variants in each category is indicated as part of the legend of each panel. Data shown are collected from ten replicates with 256 causal variants and 0.5 heritability in each species.

The characteristics of causal variants identified by BayesCπ and FarmCPU were also different. Using data from simulations conducted with 256 causal variants and heritability of 0.5, causal variants were classified into four mutually exclusive categories in each population: those identified by both methods, those identified by either only FarmCPU or only BayesCπ, and those missed by both. As shown in Figure 6, causal SNPs identified by both methods tended to have higher MAFs and larger effect sizes. SNPs identified only by FarmCPU tended to have lower MAFs than those identified only by BayesCπ in all four species (Table S2, Figure 6). However, we did not observe a statistically significant difference in effect size distribution (Table S2, Figure 6). Similar results were obtained in other levels of heritability (Figure S15). Notably, FarmCPU controlled type I error rates equivalently for SNPs of varying MAFs, while the type I error rate for BayesCπ was higher for common allelic variants and lower for rare allelic variants (Figure S16). Overall, the two approaches appear to have complementary strengths for identifying different subsets of allelic variants missed by conventional MLM‐based GWAS methods.

### Using BayesCπ to estimate genetic architecture of complex traits

**Figure 7:**
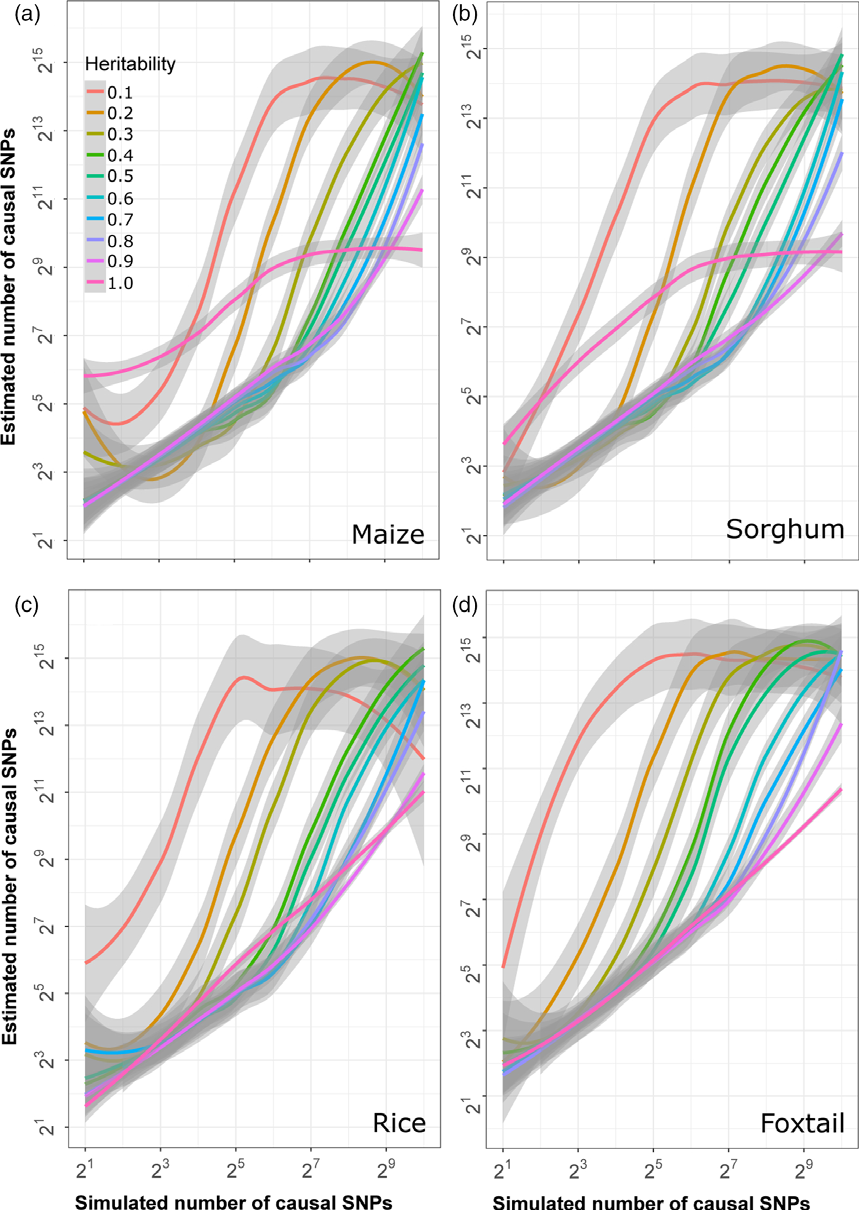
Relationship between estimated complexity of genetic architectures generated by BayesCπ and true genetic architecture complexity given different levels of heritability in each species. Grey areas indicate 95% confidence bands around each estimate.

One key difference between the GWAS presented above and real‐world GWAS is that here the complexity of the genetic architecture of each trait is a known variable. However, in real‐world application, the complexity of the genetic architecture controlling different traits may not be known prior to the start of analysis. The BayesCπ method includes a statistical approach to estimate the number of causal variants controlling a given trait prior to fitting a model to the data (Habier *et al*., 2011). These estimates serve as a prior for model fitting in BayesCπ. As different GWAS approaches provide the most favourable results for traits with different complexities, estimation of the number of genetic loci controlling a trait can also guide which GWAS approach is best suited to analyse a given dataset. The accuracy of the estimates of the number of causal variants generated by the BayesCπ approach was evaluated across varying levels of heritability and trait complexity. In all four crop species, BayesCπ was able to accurately estimate heritability for traits controlled by different numbers of causal variants (Figure S17). It also provided accurate and unbiased estimates of the number of causal variants when the heritability of the trait was high and/or the total number of causal variants was small (Figure 7). However, the number of causal variants was systematically overestimated for complex traits with lower levels of heritability (Figure 7).

## Discussion

In this study, we employed four genotype datasets with different population structures from different crop species. The MLM‐based approach showed substantial reductions in power as the complexity of the genetic architecture of the trait being analysed increased. Compared to the MLM‐based approach, FarmCPU approach and BayesCπ adopted from genomic prediction show complementary strengths, higher power and lower false discovery rates for complex traits. FarmCPU provided a more favourable trade‐off between power and FDR for moderately complex traits and a greater likelihood of identifying rare causal variants, while BayesCπ approach provided greater power to detect more causal variants with small effect sizes for extremely complex traits. However, this outperformance is less apparent or nonexistent for traits with lower levels of heritability. Present statistical approaches to GWAS have the greatest statistical power to identify SNPs which are both common, and control a large proportion of total genetic variation in the target populations. As a result, few previously unknown loci with utility for plant breeding have been discovered through GWAS‐based analysis (Bernardo, 2016). The identification of common alleles with moderate effect sizes and rare alleles with large effects would improve the utility for GWAS for both basic biological and applied applications.

Our results also indicate estimates of the complexity of the genetic architectures are clearly also needed, given the differences in the relative strengths of MLM, FarmCPU and Bayesian approaches. BayesCπ can accurately estimate the trait heritability and the number of causal variants in most situations. However, it tends to overestimate the number of causal variants for complex traits with lower levels of heritability. One potential explanation for this observation is that the model is attempting to explain residual error – not heritable phenotypic variation – by including additional, noncausal SNPs in the model. However, with awareness of this limitation, estimation of the number of causal variants controlling a given trait can aid researchers in determining, which GWAS method is likely to provide the most informative result for a given dataset.

Evaluations of GWAS approaches can be performed using either real data or simulated data. Here, simulated phenotypic dataset was employed, as it provided comprehensive information for comparison across methods, something unavailable for real‐world phenotype datasets for complex traits. The use of real‐world genotype datasets captured the patterns of MAFs, LD decay and population structures are comparable to those observed in the real world. However, it is also important to acknowledge the limitations of simulation‐based studies. The simulated phenotype datasets employed here assumed the effect sizes of minor alleles are drawn from a normal distribution, which is supported by real‐world observations of multiple complex traits as shown in Figure S18 (Brown *et al*., 2011; Buckler *et al*., 2009). Notably, the comparison of MLM and FarmCPU here are similar to the results described in (Liu *et al*., 2016), which employed a geometric distribution of effect sizes. However, not all traits will exhibit a normal distribution of effect sizes for underlying genetic loci. For example, traits which have experienced strong and recent natural or artificial selection are likely to exhibit a non‐normal distribution of effect sizes (Orr, 1998; Wallace *et al*., 2014; Xu *et al*., 2017) and the absolute estimates of power presented here are likely to be inflated for such traits. More significantly, the simulation parameters used assumed no correlation between the minor allele frequency of an allele and its effect size, which does not match predictions from population genetic theory or observation that rare alleles tend to be associated with larger molecular phenotypes in maize (Kremling *et al*., 2018). These simulations assumed that the true functional variant was included as one of the genotyped markers within the dataset. At the moment, in many populations the best case outcome for researchers is to identify a genetic marker in high LD with the true causal variant. In the future whole‐genome resequencing or independent genome assemblies may make the identification of true causal variants more likely, at least in species such as maize which exhibit rapid LD decay. In addition, the statistical model used to generate phenotype datasets here did not incorporate epistatic interactions between causal variants.

While the results presented here for the use of BayesCπ to identify causal variants are promising, additional work is needed to further adapt BayesCπ for use in GWAS applications. The model employed here did not yet incorporate any controls for population structure which may explain a portion of the higher type one error rates observed for this method. Integrating such a control might marginally reduce power. The model we employed provided a ranking of genetic markers but not the straightforward method of establishing a cut‐off between candidate causal variants and noncandidate loci. Although ranking enabled comparisons of power, type I error, and false discovery rate, the application of BayesCπ‐based GWAS in a real‐world setting will require methods to establish such cut‐offs. One promising approach recently discussed in the literature is to estimate posterior type I error rates (Fernando *et al*., 2017). Approaches using machine learning to identify cut‐offs, such in NeuralFDR, also seem a promising avenue of investigation (Xia *et al*., 2017). In addition, computational resource requirements play a substantial role in which statistical approaches become widely adopted over time. With the largest of the four genotype datasets employed here (maize) BayesCπ required approximately 4.5 Gb of RAM and 2 h to analyse one dataset. For comparison, the MLM implementation in GEMMA required only 1 Gb of RAM and approximately 40 min to analyse the same dataset and FarmCPU required approximately 30 min and 5.5 Gb RAM (Figures S19 and S20). However, optimisation of computational pipelines can reduce run times dramatically without the need for changes to statistical models. For example, modifications to the reference implementation of the FarmCPU algorithm have been shown to produce the same results while reducing runtime by approximately two‐thirds (Schnable and Kusmec, 2017).

## Conclusion

Association studies have been and seem likely to remain an important tool for investigating how genotype determines phenotype. Although certain diseases and target traits for breeding efforts are controlled by a small number of large effect loci segregating in Mendelian fashion, many traits of interest are controlled by moderately or extremely complex genetic architectures. Here, we have shown that different approaches to GWAS have complementary strengths, and the complexity of the genetic architecture controlling a target trait should be determined prior to the selection of an appropriate statistical approach for analysing a given dataset. Further improvements in both statistical approaches and computational optimisation hold the promise of dramatically expanding our understanding of the role that both rare alleles with large consequences and common alleles with small consequences play in determining how genotype determines phenotype across species.

## Methods

### Genotype dataset sources and filtering parameters

Genotype dataset for foxtail millet (*Setaria italica*) (Jia *et al*., 2013), maize (*Zea mays*) (Romay *et al*., 2013), sorghum (*Sorghum bicolor*) (Lasky *et al*., 2015), and rice (*Oryza sativa*) (McCouch *et al*., 2016) were taken from published sources. Foxtail millet SNPs were discovered and scored using low coverage (0.5×) whole‐genome resequencing reads aligned to the *Setaria italica* reference genome (v2 from Phytozome v7.0; Bennetzen *et al*., 2012). The partially imputed SNP dataset was downloaded from Millet GWAS Project website (http://202.127.18.221/MilletHap1/GWAS.php). The downloaded genotype data included 916 diverse varieties and 726 080 SNPs with minor allele frequencies lower than 5% had been removed prior to the publication of the dataset. After downloading, SNPs without calls in >10% of samples were removed from the dataset. The sorghum GBS dataset which included 404 627 SNPs scored relative to the v1.4 of the sorghum reference genome (Paterson *et al*., 2009) across a set of 1943 accessions were downloaded from Data Dryad website (http://datadryad.org/resource/doi:10.5061/dryad.jc3ht/1; Lasky *et al*., 2015). The maize GBS dataset which included calls for 681 257 SNPs relative to B73 RefGenV1 (Schnable *et al*., 2009) across a set of 2815 accessions was downloaded from Panzea website (https://www.panzea.org/; Romay *et al*., 2013). After downloading, both SNPs in sorghum and maize without genotype calls in >30% of samples and SNPs with heterozygous calls in >5% of samples were removed from the datasets. After any filtering parameters described above for individual datasets, missing data in foxtail millet, sorghum and maize dataset were imputed using Beagle v4.1 with default parameters (Browning and Browning, 2016). Data from genotyping 1568 diverse rice accessions using the 700 000 marker HDRA microarray platform were downloaded from GEO (ID: GSE71553) (McCouch *et al*., 2016). The downloaded SNPs with heterozygous genotype calls in >5% of samples were removed. Statistics on the final number of SNP markers and samples in each dataset are provided in Table 1.

### Characteristics and summary statistics of genotype datasets used in this study

The minor allele frequency was calculated for each SNP in each dataset. Patterns of minor allele frequency distributions for each dataset were assessed and visualized using kernel density plots generated using the function ‘kdeplot’ from the Python package ‘seaborn’. For each dataset, the top ten principal components were calculated using Tassel (version 5.0; Bradbury *et al*., 2007). The top three principal components from the same analyses were used to plot population structure using the R package scatterplot3d. Plink 1.9 was used to calculated *r* ^2^ between all pairs of SNP markers separated by less than 10 million bases (Purcell *et al*., 2007). The average *r* ^2^ values were calculated from 10^0.1^ to 10^7^ using a logarithmic step size of 0.1. A regression curve was fit to these values using the function ‘regplot’ from the Python package ‘seaborn.’

### Phenotype simulation

Phenotype datasets were simulated using an additive genetic model (Equation (1)) derived from the underlying genotype datasets.

(1) $$Y_{j}=\sum \left(a_{i}\times S_{\mathrm{ij}}\right)+e_{j}$$

In the model, *Y*_*j*_ is the simulated phenotype for plant *j*;* a*_*i*_ is the effect of the *i‐*th causal SNP; *S*_*ij*_ is the SNP genotype (coded with 0, 1, 2) for the *i‐*th causal SNP of the *j‐*th plant; and *e*_*j*_ is the residual error for *j*‐th plant extracted from a normal distribution with mean of 0 and standard deviation of $\frac{\text{Var}\left(\sum S_{\mathrm{ij}}a_{i}\right)}{1/h^{2}-1}$, where *h* ^*2*^ denotes the heritability.

An R function ‘simcrop’ was implemented within the open source ‘g3tools’ R package (https://github.com/jyanglab/g3tools). For the results employed in this study, both the effect sizes of individual SNPs *a*_*i*_ and the error term in measurements of individual genotypes *e*_*j*_ were drawn from normal distributions. However, the software package developed to enable this study also provides the option to specify other effect size distribution models. Phenotype datasets were simulated for scenarios where the number of causal genetic loci ranged from 2^1^ to 2^10^ (2–1024 QTNs) and where the heritability of trait values ranged from 0.1 to 1.0 in steps of 0.1. For each combination of heritability and number of causal variants, 10 independent replicates with different randomly selected causal variants were generated.

### Methods of Genome‐wide association studies

All MLM‐based GWAS analysis in this study were performed using GEMMA (version 0.95alpha) with the command “gemma ‐g [genotype mean file] ‐a [genotype annotation file] ‐p [phenotype file] ‐c [PCs file] ‐k [kinship file] ‐o [output file]” (Zhou and Stephens, 2012). Tassel (version 5.0) was used to generate PCs and the first three PCs from the Tassel analysis were included in both the MLM and FarmCPU analyses (Bradbury *et al*., 2007). The kinship matrix file applied in MLM method was generated using “gemma ‐gk 1” command in GEMMA package for each genotype dataset. Within the MLM, population structure (Q) and the relationship among individuals (K) were fitted at the same time, which is also called as *Q* + *K* model: *y* = *Q* + *K* + *s* + *e*, where *y* is an vector of phenotype values for all the individuals in the population and *e* is the residue; *Q* is a matrix known as covariates/PCs representing the population structure; *K* is the kinship matrix representing the relationship among individuals; and *s* is the genetic effects.

FarmCPU was run using the command: FarmCPU (Y = myY, GD = myGD, GM = myGM, CV = myCV, method.bin = “optimum”) in R. Y, GD and GM represent phenotype, genotype and genotypic map data respectively. CV represents the principal components file. The kinship matrix was automatically estimated in FarmCPU. While kinship matrices were independently generated by GEMMA and FarmCPU, the correlation between these two matrices is over 0.9999 (Pearson *r* ^2^). The parameter method.bin = “optimum” allows the FarmCPU to selected optimized possible QTN window size and number of possible QTNs in the model (Liu *et al*., 2016).

The BayesCπ approach was adopted from genomic prediction area to represent Bayesian multiple‐regression methods for GWAS. BayesCπ was conducted using GenSel software package (Version 2.14; Habier *et al*., 2011). In the Bayesian method, a two‐step procedure was employed to account for the potential effects of the arbitrary priors (Yang *et al*., 2018). In the test run, 1000 iterations was used with 100 burn‐in iterations of MCMC simulations using default priors: genetic variance = 1 and residual variance = 1. In the real run, the priors were replaced using the posteriors obtained from test run and a longer chain of simulations was employed (chain length = 11 000, burnin = 1000 and π = 0.9999). All the GWAS jobs were run on HCC's (the Holland Computing Center) Crane cluster at University of Nebraska‐Lincoln.

### Statistical evaluation of accuracy and power

For all genetic markers assigned *P*‐values by GEMMA or FarmCPU, individual markers were sorted by reported *P*‐values. Within BayesCπ, markers were first sorted by model frequency. When multiple markers were assigned the same model frequency by BayesCπ, ties were broken by the genetic variance assigned to each marker by BayesCπ. To evaluate FDR, statistical power and Type I error, an increasing rank method was applied. Each rank contains the first *K* markers from the sorted GWAS results as described above. These *K* markers were treated as positive markers and rest of markers were treated as negative markers. A true positive marker was defined if it was in the causal variants list or exhibited LD *r* ^2^ **> 0.6 with a causal variant. LD thresholds between 0.6 and 0.9 did not significantly change observed results (Figure S21), while considering only causal variants as true positives significantly decreased power for all methods. If there several positive markers were linked to the same causal variants, the combined set was counted as only a single true positive and the number of total positives reduced accordingly. False positives were defined as positives neither in causal variants list nor in LD with causal variants. For each scenario, power was defined by Equation (2):

(2) $$\text{Power}=\frac{\text{No. of true positive SNPs}}{\text{No. of total causal SNPs}}$$

The corresponding FDR and Type I error were defined by Equation 3 and 4:

(3) $$\text{FDR}=\frac{\text{No. of flase positive SNPs}}{\text{No. of positive SNPs}}$$

(4) $$\text{Type I error}=\frac{\text{No. of false positive SNPs}}{\text{No. of noncausal SNPs}}$$

## Author's contributions

JS designed the project. JY generated phenotype datasets and conducted the BayesCπ analysis. CM conducted all other analysis. CM, JY and JS generated figures and wrote the manuscript. All authors have read and approved the final manuscript.

## Conflict of interest

The authors have declared that no competing interests exist.

## Supporting information


**Figure S1** Relationship between LD decay and outcrossing rates reported from the literature for maize, sorghum and foxtail millet.
**Figure S2** Relationship between the proportion of causal variants identified and heritability for traits controlled by different numbers of causal variants (2–1024 causal variants) in each species in an MLM‐based GWAS.
**Figure S3** Relationship between the proportion of causal variants identified and the number of causal variants controlling a trait given different levels of heritability (0.1–1) in each species in an MLM‐based GWAS.
**Figure S4** Distribution of minor allele frequency and effect size for true positive and false negative causal variants in each species.
**Figure S5** Relationship between the proportion of causal variants identified and the number of associated SNPs selected for MLM, FarmCPU and Bayesian analysis for 4, 8 and 32 causal variants.
**Figure S6** Relationship between the proportion of causal variants identified and the number of associated SNPs selected for MLM, FarmCPU and Bayesian analysis for 128, 512 and 1024 causal variants.
**Figure S7** Relationship between false discovery rate and the number of associated SNPs selected for MLM, FarmCPU and Bayesian analysis for 4, 8 and 32 causal variants.
**Figure S8** Relationship between false discovery rate and the number of associated SNPs selected for MLM, FarmCPU and Bayesian analysis for 128, 512 and 1024 causal variants.
**Figure S9** Relationship between the proportion of causal variants identified and the number of associated SNPs selected for MLM, FarmCPU and Bayesian analysis for 16, 64 and 256 causal variants.
**Figure S10** Relationship between the proportion of causal variants identified and the number of associated SNPs selected for MLM, FarmCPU and Bayesian analysis for 16, 64 and 256 causal variants.
**Figure S11** Relationship between false discovery rate and the number of associated SNPs selected for MLM, FarmCPU and Bayesian analysis for 16, 64 and 256 causal variants.
**Figure S12** Relationship between false discovery rate and the number of associated SNPs selected for MLM, FarmCPU and Bayesian analysis for 16, 64 and 256 causal variants.
**Figure S13** Relationship between false‐positive rate (Type I error) and the number of associated SNPs selected for MLM, FarmCPU and Bayesian analysis for 4, 8 and 32 causal variants.
**Figure S14** Relationship between false‐positive rate (Type I error) and the number of associated SNPs selected for MLM, FarmCPU and Bayesian analysis for 16, 64 and 256 causal variants.
**Figure S15** Differences in the characteristics of causal SNPs identified by BayesCπ and FarmCPU. Distribution of MAF (Left) and absolute effect size (Right) for causal variants identified by both BayesCπ and FarmCPU, only BayesCπ, only FarmCPU, or neither approach.
**Figure S16** Relationship between minor allele frequency and type I error rates for markers in the maize, sorghum and rice datasets for both FarmCPU and BayesCπ.
**Figure S17** Relationship between simulated heritability and heritability estimates generated by BayesCπ for traits controlled by different numbers of causal variants.
**Figure S18** Empirically determined effect sizes for loci control seven different traits in maize.
**Figure S19** Average run time of a single GWAS analysis using each of the three methods evaluation in each of the four populations tested.
**Figure S20** Average maximum memory use of a single GWAS analysis using each of the three methods evaluation in each of the four populations tested.
**Figure S21** The influence of different LD decay cut‐offs on apparent GWAS power. Data shown are from simulations where the number of causal variants is 32 and heritability is 0.7.

**Table S1**. Mann–Whitney *U* test between SNP groups detected and undetected by GEMMA. Data shown are from simulations where the number of causal variants is 64 and heritability is 0.7.
**Table S2**. Mann–Whitney *U* test between SNP groups identified by FarmCPU, BayesCπ, both and neither. Number of causal SNPs is 256 and heritability is 0.5.

**Appendix S1** The significant SNPs identified by MLM, FarmCPU and BayesCπ in real‐world maize flowering time dataset.
