# Supplementary material for: Optimising the identification of causal variants across varying genetic architectures in crops
Source: Plant Biotechnol J. 2018 Nov 9;17(5):893–905. doi: 10.1111/pbi.13023 (PMC6587547; doi:10.1111/pbi.13023)
Supplement: Supplementary file 1 — Figure S1 Relationship between LD decay and outcrossing rates reported from the literature for maize, sorghum and foxtail millet. Figure S2 Relationship between the proportion of causal variants identified and heritability for traits controlled by different numbers of causal variants (2–1024 causal variants) in each species in an MLM‐based GWAS. Figure S3 Relationship between the proportion of causal variants identified and the number of causal variants controlling a trait given different levels of heritability (0.1–1) in each species in an MLM‐based GWAS. Figure S4 Distribution of minor allele frequency and effect size for true positive and false negative causal variants in each species. Figure S5 Relationship between the proportion of causal variants identified and the number of associated SNPs selected for MLM, FarmCPU and Bayesian analysis for 4, 8 and 32 causal variants. Figure S6 Relationship between the proportion of causal variants identified and the number of associated SNPs selected for MLM, FarmCPU and Bayesian analysis for 128, 512 and 1024 causal variants. Figure S7 Relationship between false discovery rate and the number of associated SNPs selected for MLM, FarmCPU and Bayesian analysis for 4, 8 and 32 causal variants. Figure S8 Relationship between false discovery rate and the number of associated SNPs selected for MLM, FarmCPU and Bayesian analysis for 128, 512 and 1024 causal variants. Figure S9 Relationship between the proportion of causal variants identified and the number of associated SNPs selected for MLM, FarmCPU and Bayesian analysis for 16, 64 and 256 causal variants. Figure S10 Relationship between the proportion of causal variants identified and the number of associated SNPs selected for MLM, FarmCPU and Bayesian analysis for 16, 64 and 256 causal variants. Figure S11 Relationship between false discovery rate and the number of associated SNPs selected for MLM, FarmCPU and Bayesian analysis for 16, 64 and 256 causal variants. Figure S12 Rel [file PBI-17-893-s002.docx]

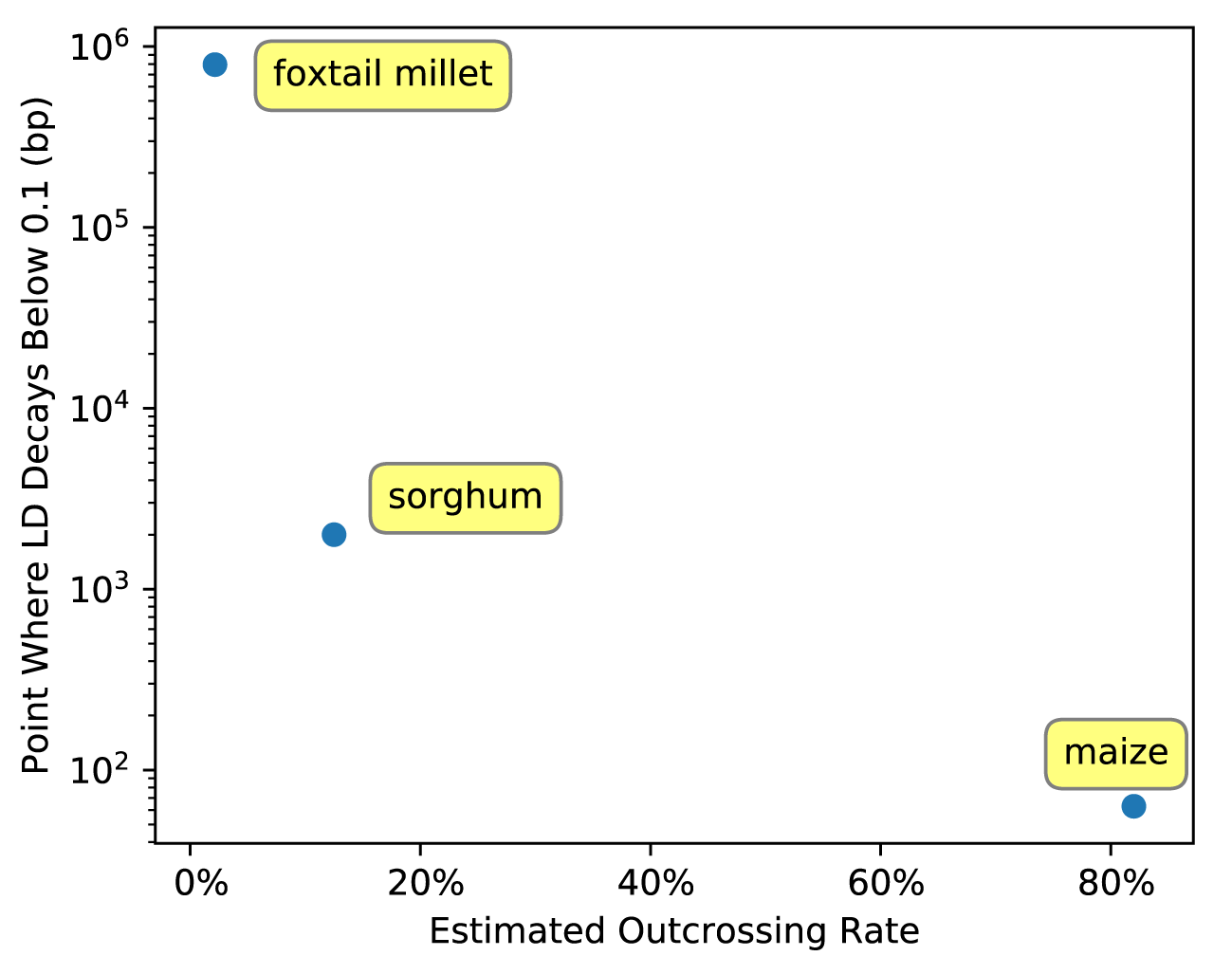


**Figure S1**. Relationship between LD decay and outcrossing rates reported from the literature for maize, sorghum, and foxtail millet. Estimated outcrossing rates for each species are taken from previous studies (Gutierrez and Sprague, 1959; Djè *et al*., 2004; Barnaud *et al*., 2008; Wang *et al*., 2010; Hufford *et al*., 2011).


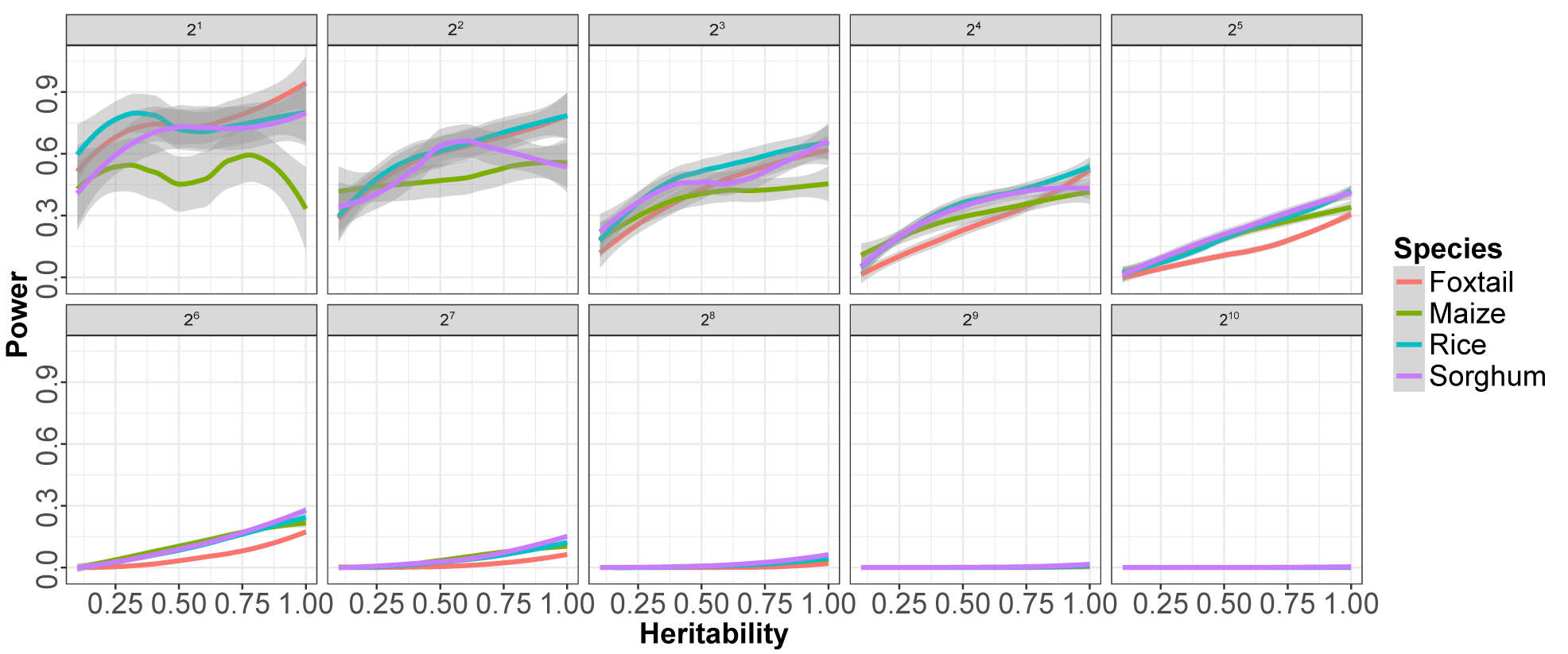


**Figure S2**. Relationship between the proportion of causal variants identified and heritability for traits controlled by different numbers of causal variants (2-1024 causal variants) in each species in an MLM-based GWAS. Positive calls were defined as those above a Bonferroni corrected *p*-value cutoff of 0.05.


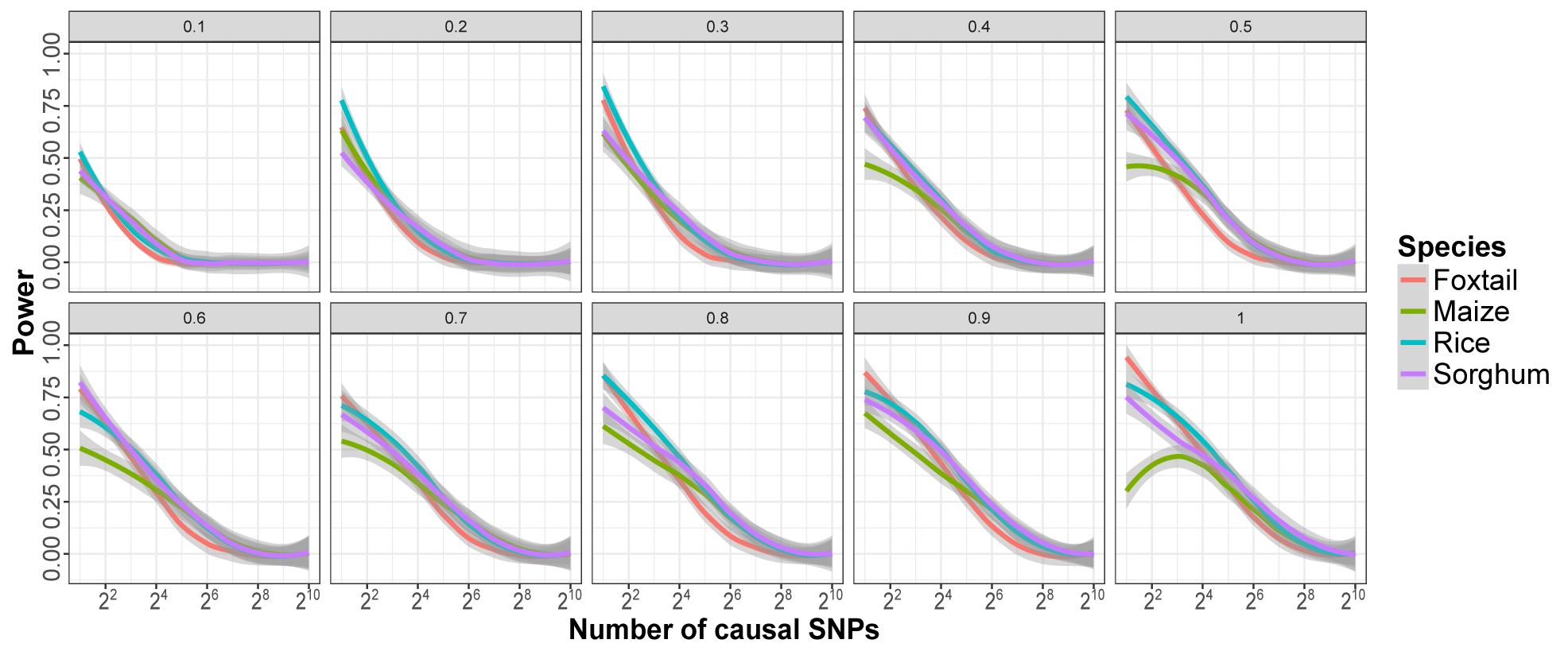


**Figure S3**. Relationship between the proportion of causal variants identified and the number of causal variants controlling a trait given different levels of heritability (0.1-1) in each species in an MLM-based GWAS. Positive calls were defined as those above a Bonferroni corrected *p*-value cutoff of 0.05.


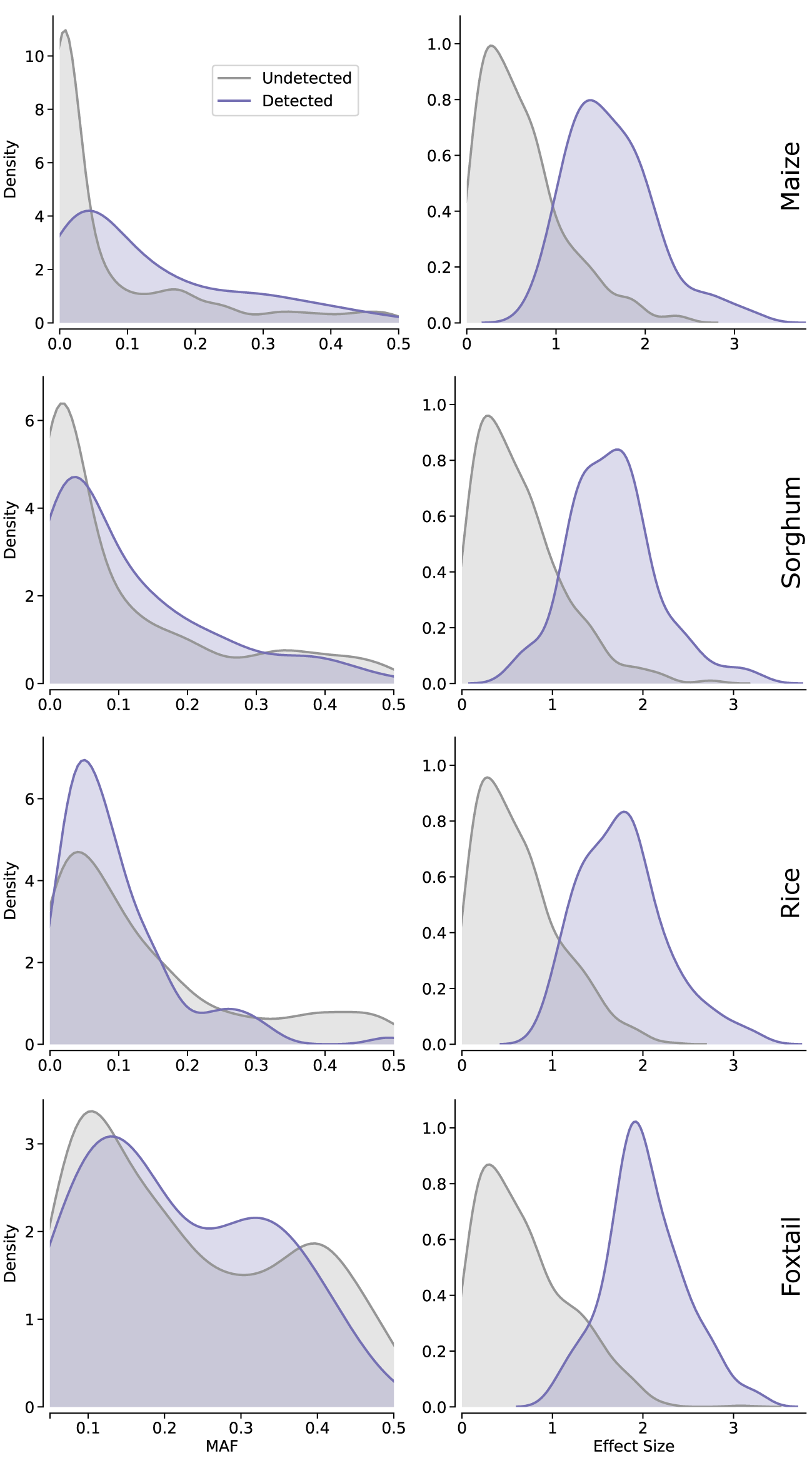


**Figure S4**. Distribution of minor allele frequency and effect size for true positive and false negative causal variants in each species. Data shown are from simulation where the heritability is 0.7 and the number of simulated causal variants is 64. Positive calls were defined as those above a Bonferroni corrected *p*-value cutoff of 0.05.


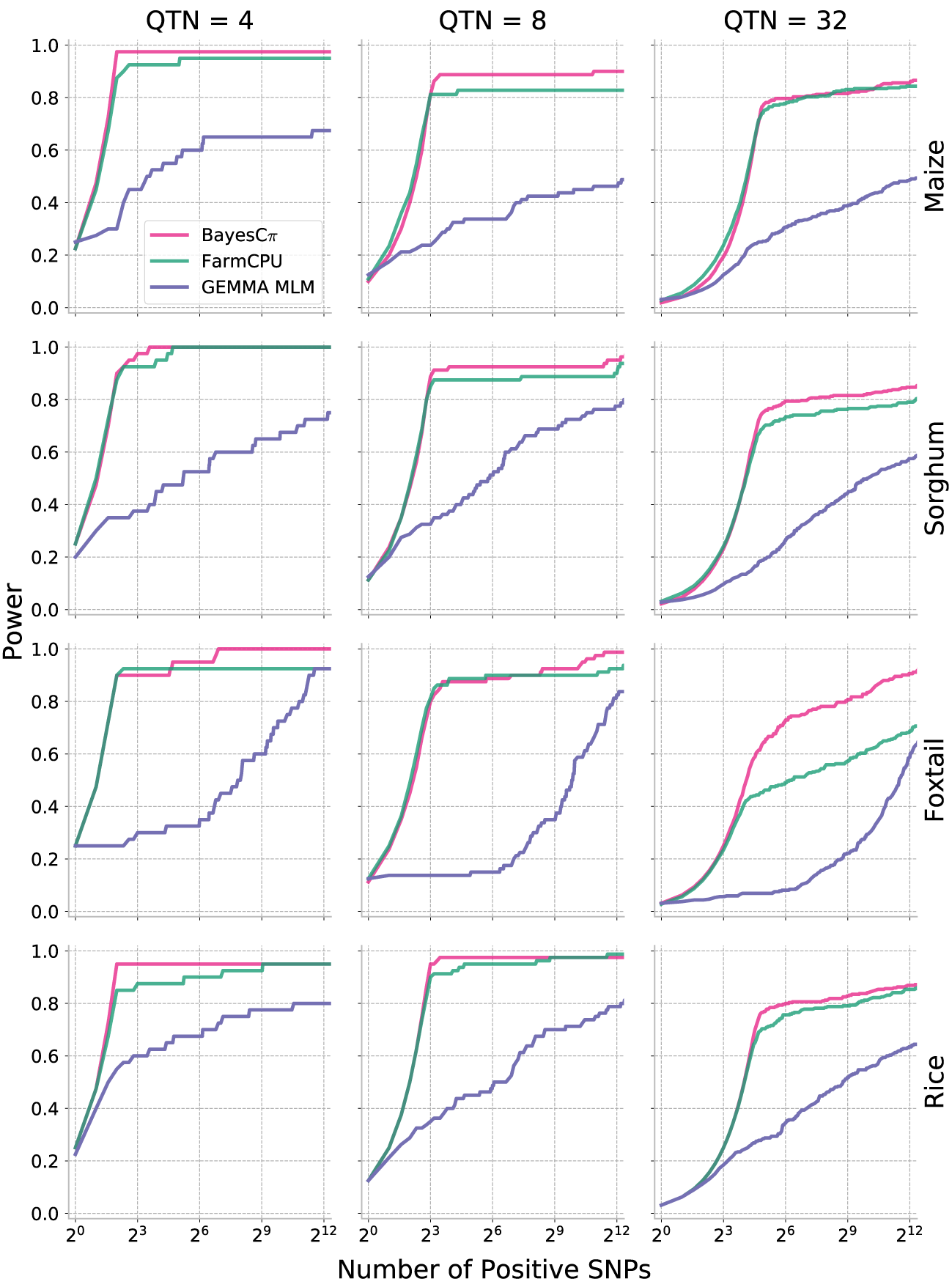


**Figure S5**. Relationship between the proportion of causal variants identified and the number of associated SNPs selected for MLM, FarmCPU, and Bayesian analysis for 4, 8, and 32 causal variants. Data shown are from simulations where trait heritability is 0.9.


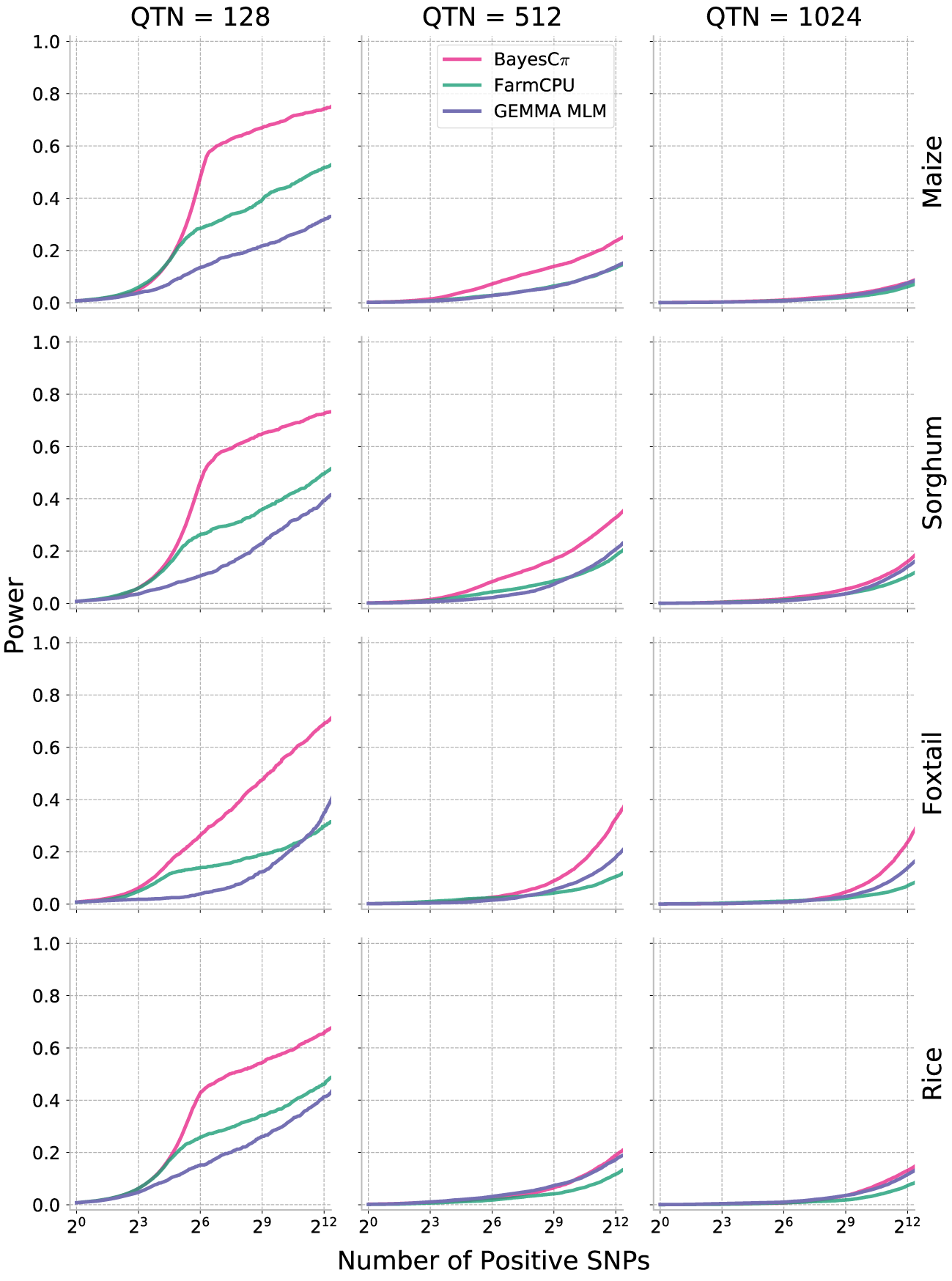


**Figure S6**. Relationship between the proportion of causal variants identified and the number of associated SNPs selected for MLM, FarmCPU, and Bayesian analysis for 128, 512, and 1024 causal variants. Data shown are from simulations where trait heritability is 0.9.


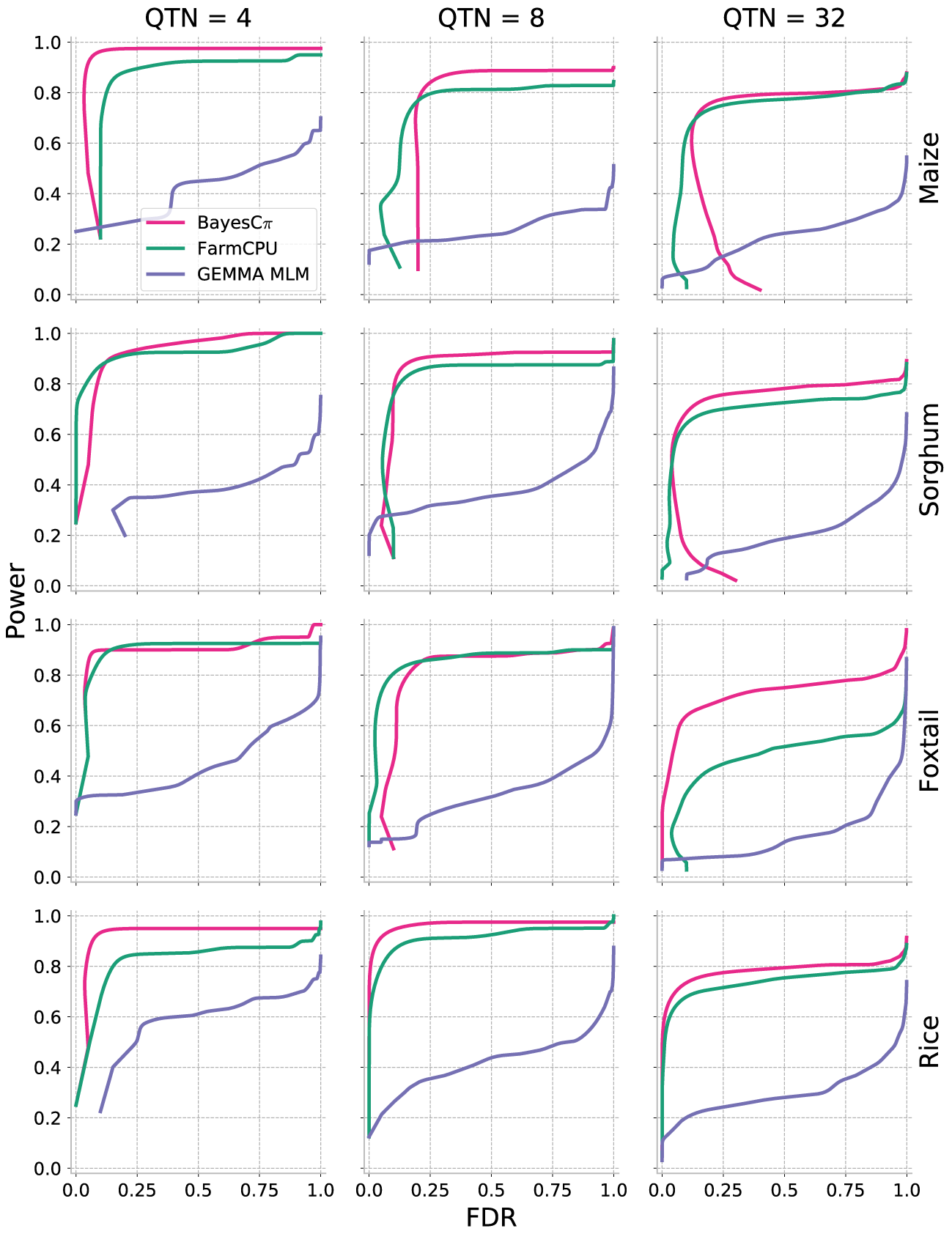


**Figure S7**. Relationship between false discovery rate and the number of associated SNPs selected for MLM, FarmCPU, and Bayesian analysis for 4, 8, and 32 causal variants. Data shown are from simulations where trait heritability is 0.9.


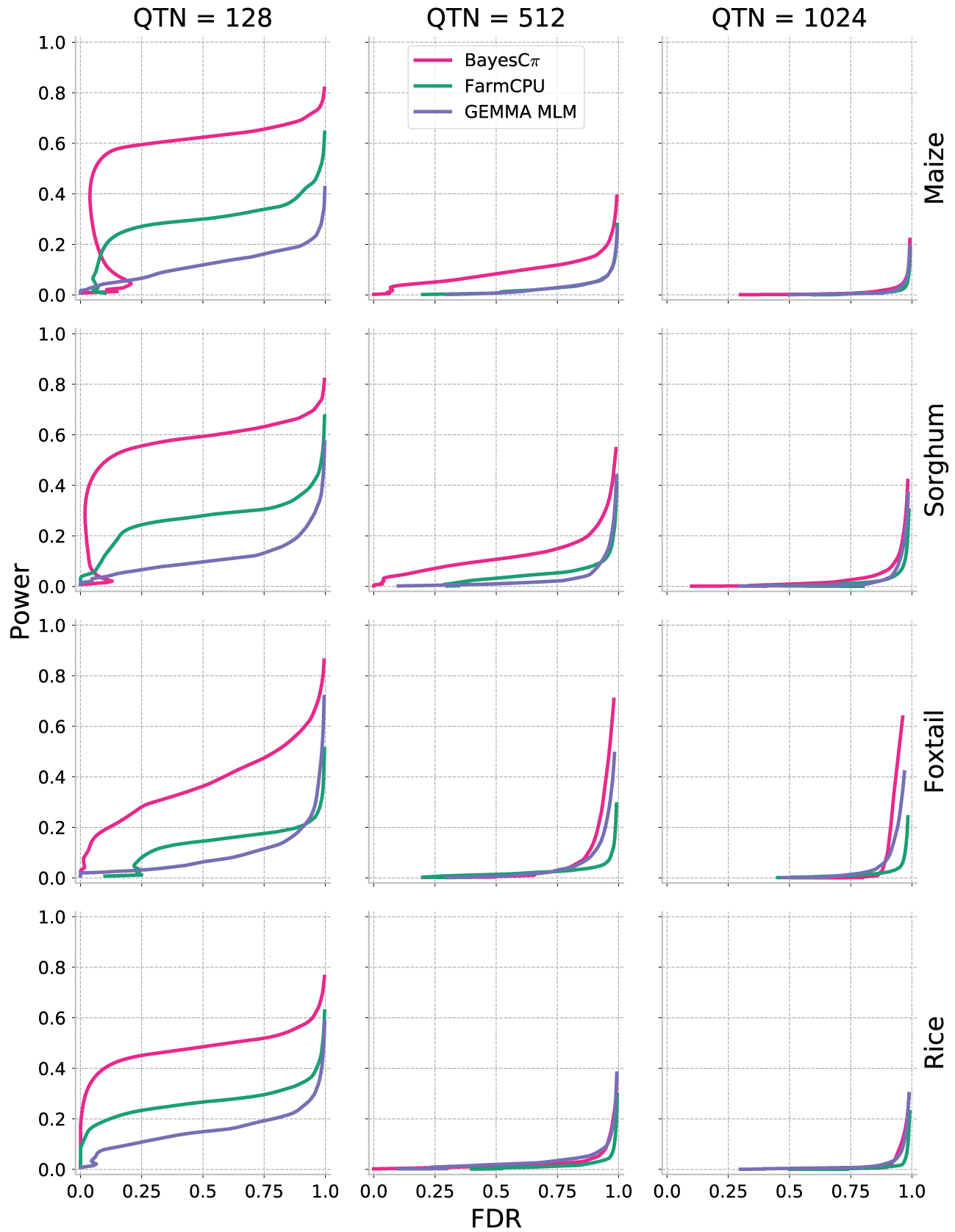


**Figure S8**. Relationship between false discovery rate and the number of associated SNPs selected for MLM, FarmCPU, and Bayesian analysis for 128, 512, and 1024 causal variants. Data shown are from simulations where trait heritability is 0.9.


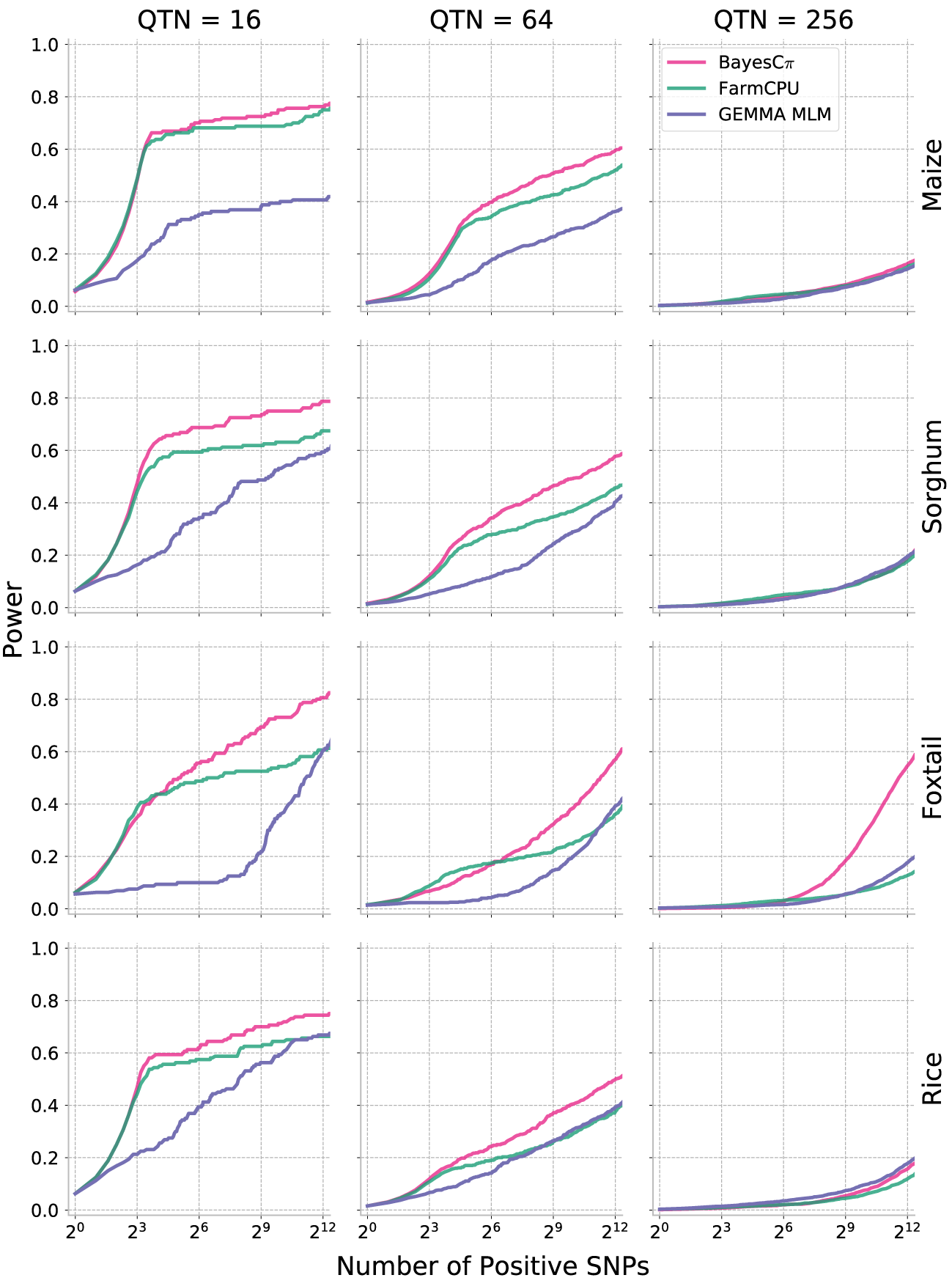


**Figure S9**. Relationship between the proportion of causal variants identified and the number of associated SNPs selected for MLM, FarmCPU, and Bayesian analysis for 16, 64, and 256 causal variants. Data shown are from simulations where trait heritability is 0.5.


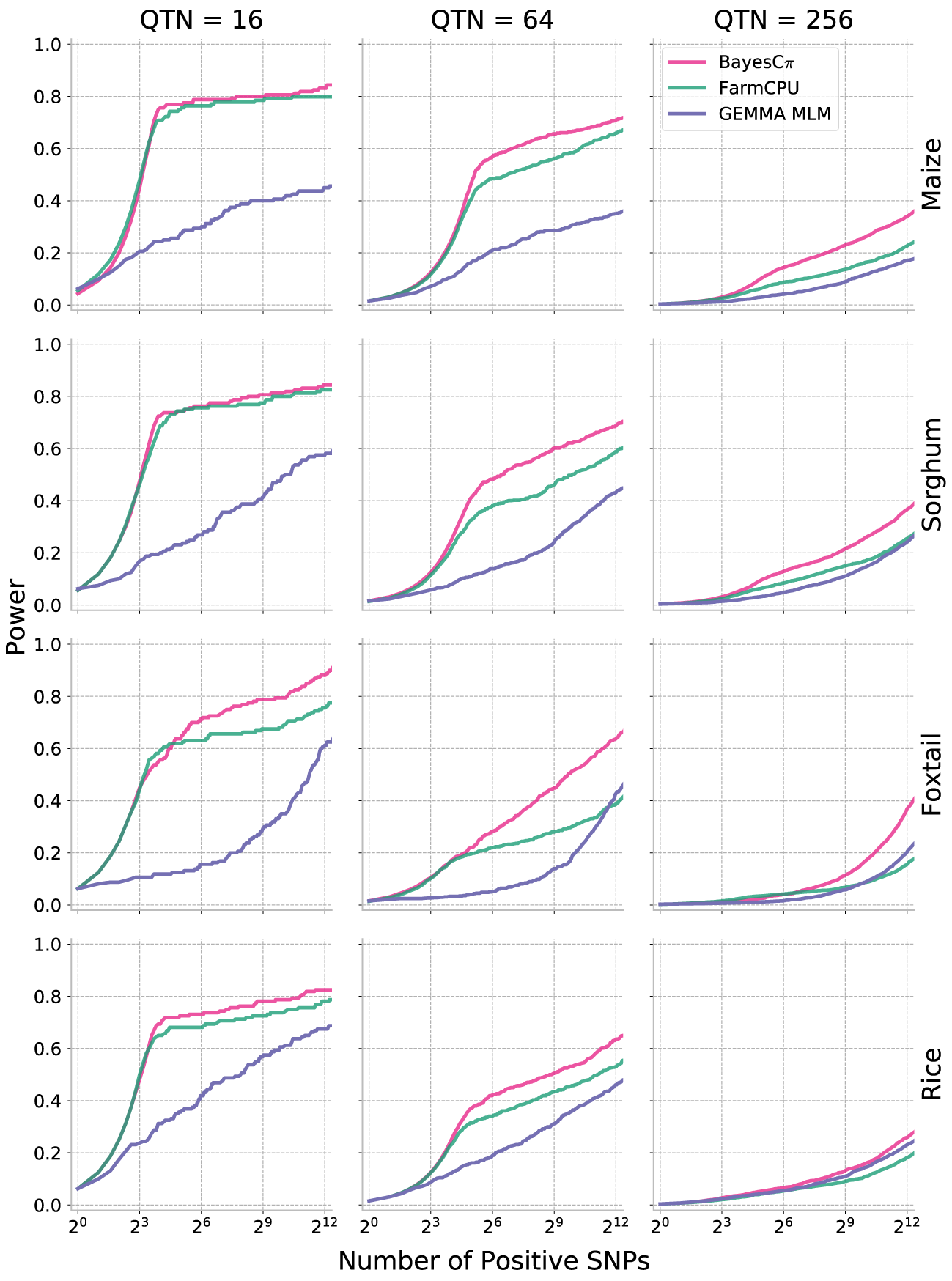


**Figure S10**. Relationship between the proportion of causal variants identified and the number of associated SNPs selected for MLM, FarmCPU, and Bayesian analysis for 16, 64, and 256 causal variants. Data shown are from simulations where trait heritability is 0.7.


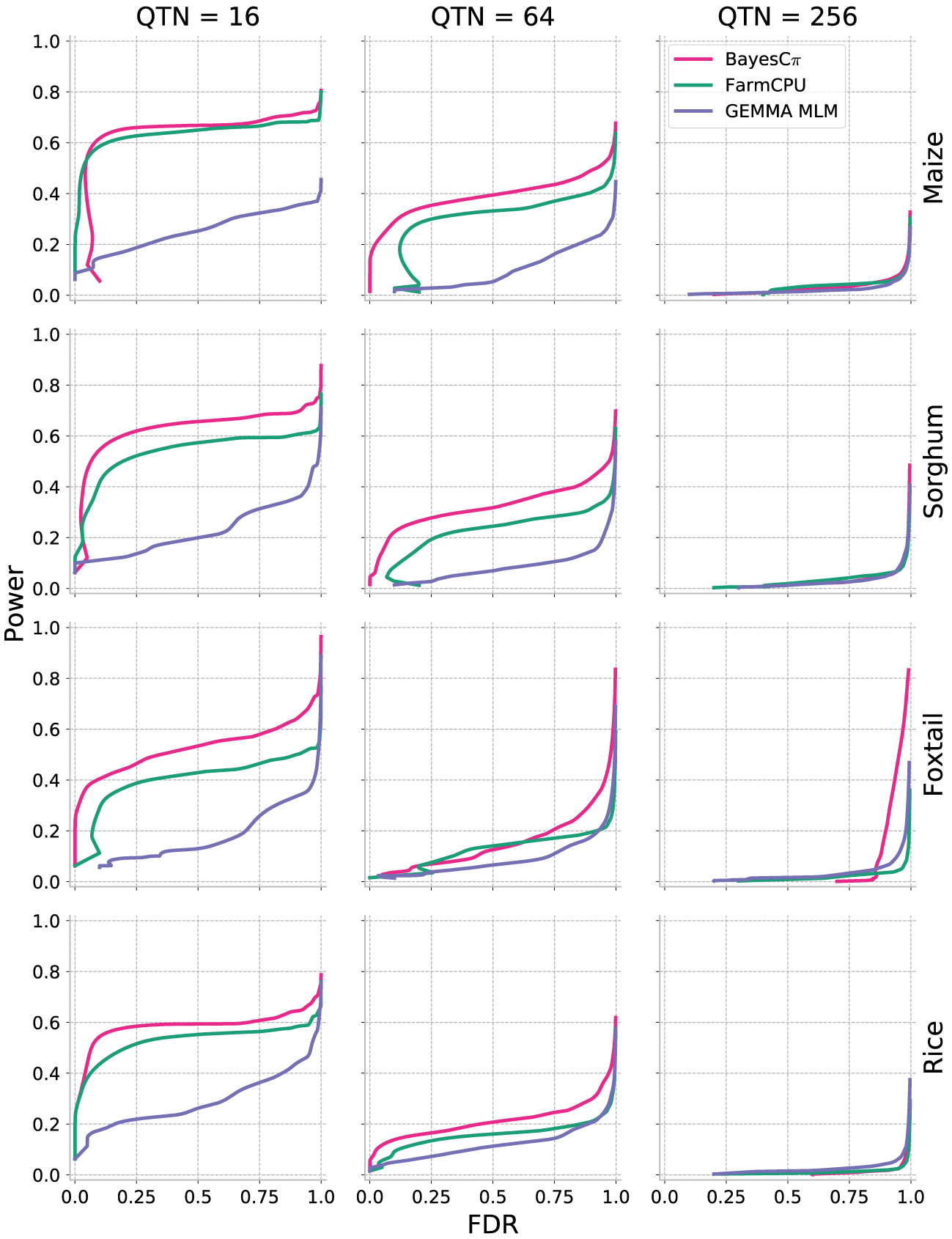


**Figure S11**. Relationship between false discovery rate and the number of associated SNPs selected for MLM, FarmCPU, and Bayesian analysis for 16, 64, and 256 causal variants. Data shown are from simulations where trait heritability is 0.5.


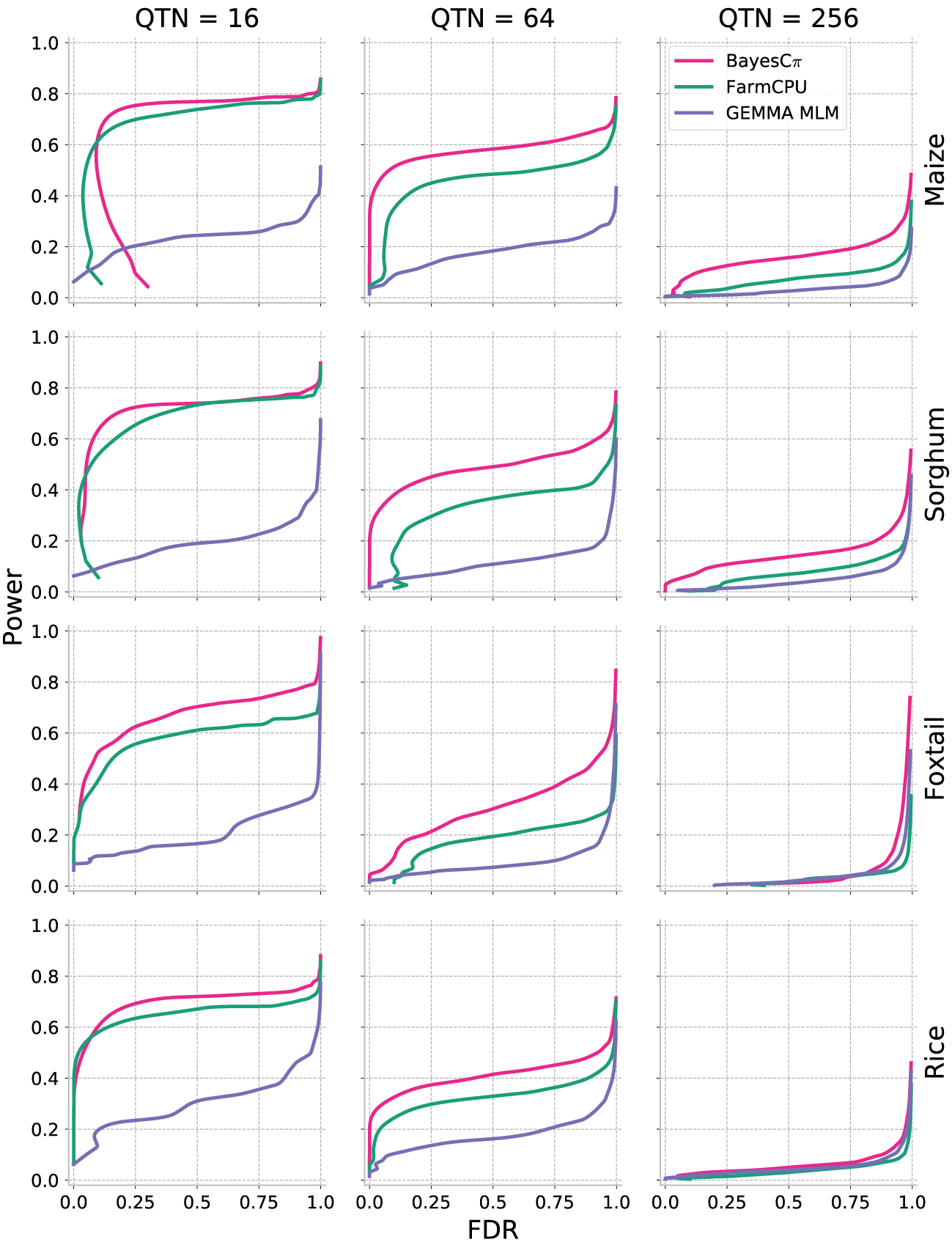


**Figure S12**. Relationship between false discovery rate and the number of associated SNPs selected for MLM, FarmCPU, and Bayesian analysis for 16, 64, and 256 causal variants. Data shown are from simulations where trait heritability is 0.7.


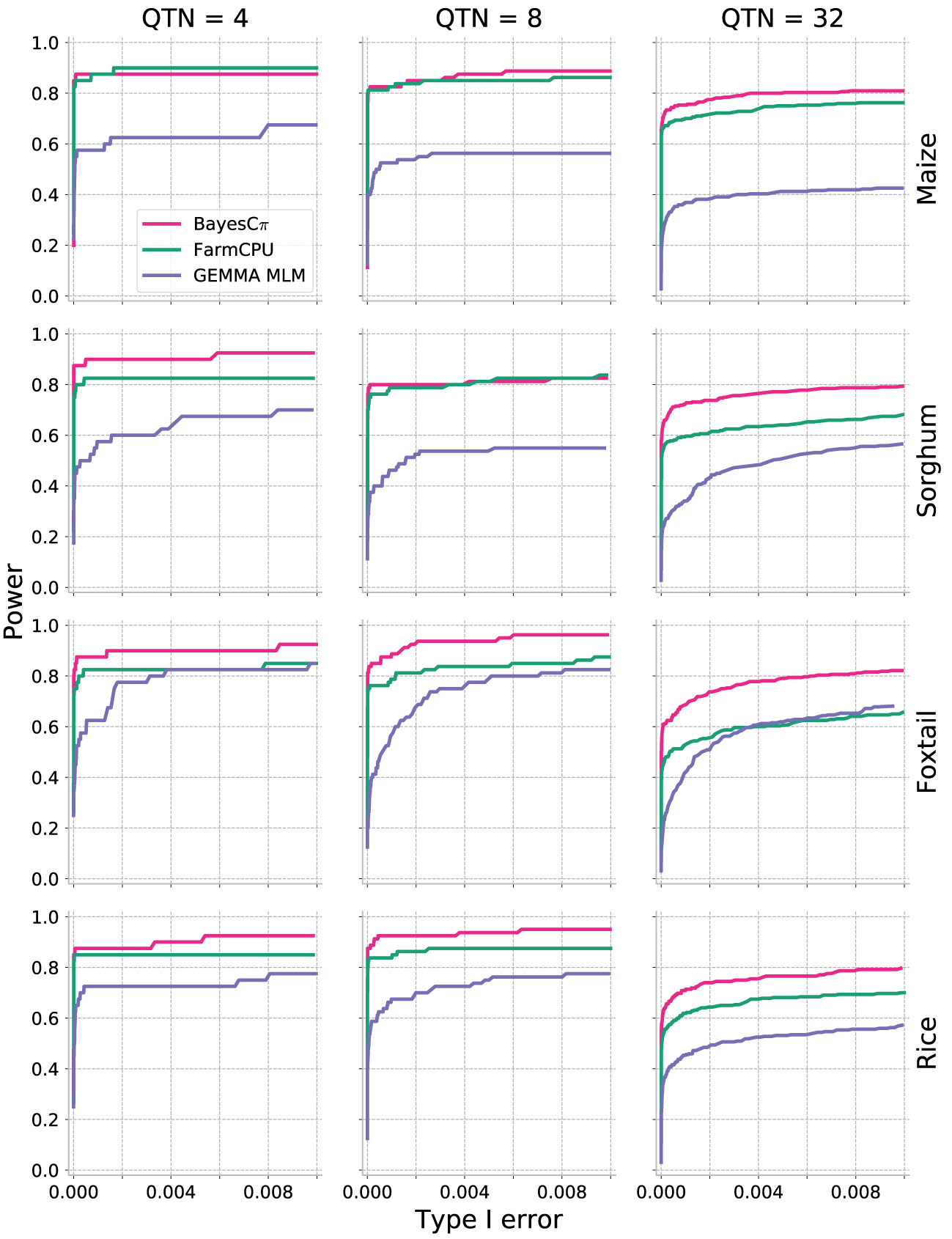


**Figure S13**. Relationship between false positive rate (Type I error) and the number of associated SNPs selected for MLM, FarmCPU, and Bayesian analysis for 4, 8, and 32 causal variants. Data shown are from simulations where trait heritability is 0.7.


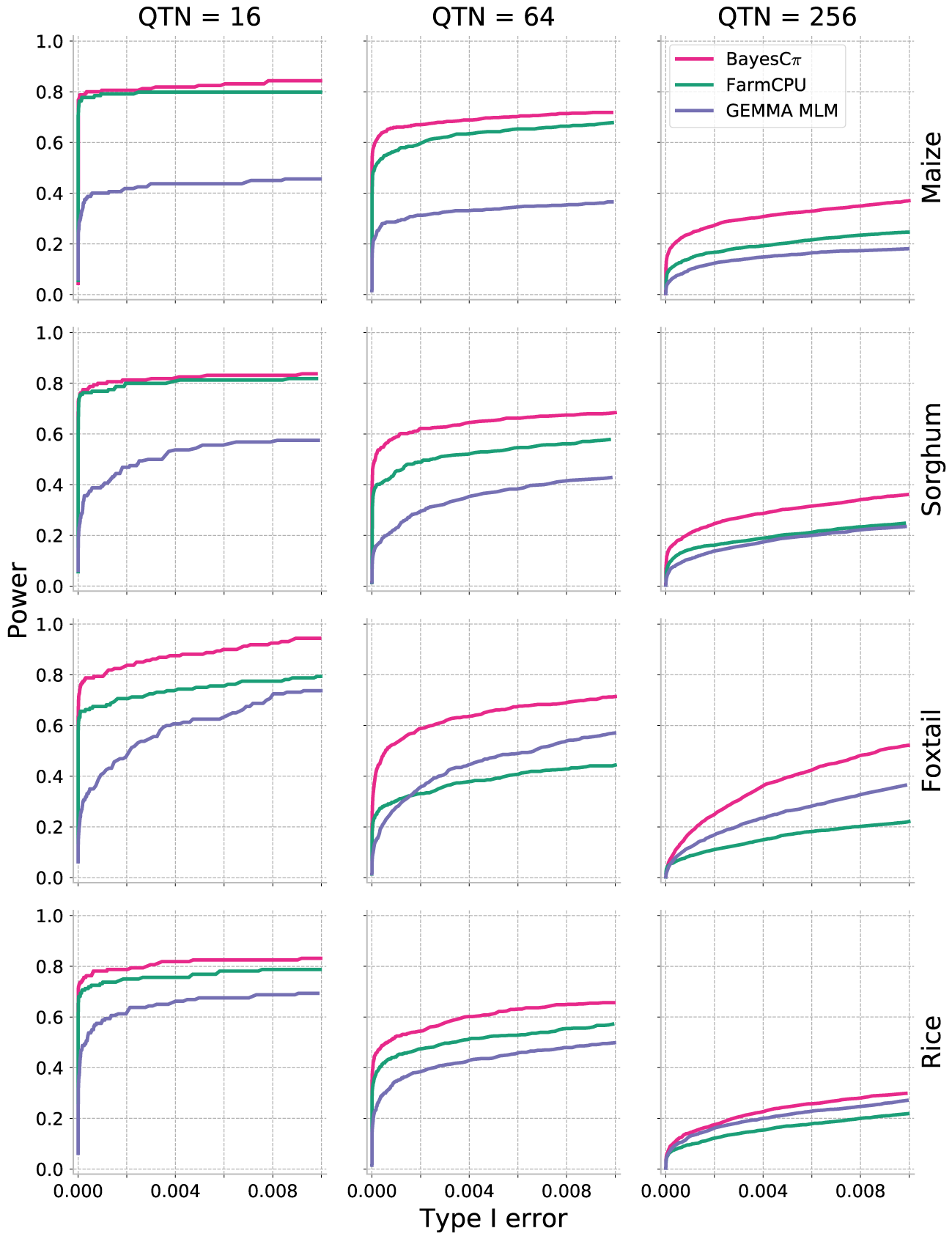


**Figure S14**. Relationship between false positive rate (Type I error) and the number of associated SNPs selected for MLM, FarmCPU, and Bayesian analysis for 16, 64, and 256 causal variants. Data shown are from simulations where trait heritability is 0.7.


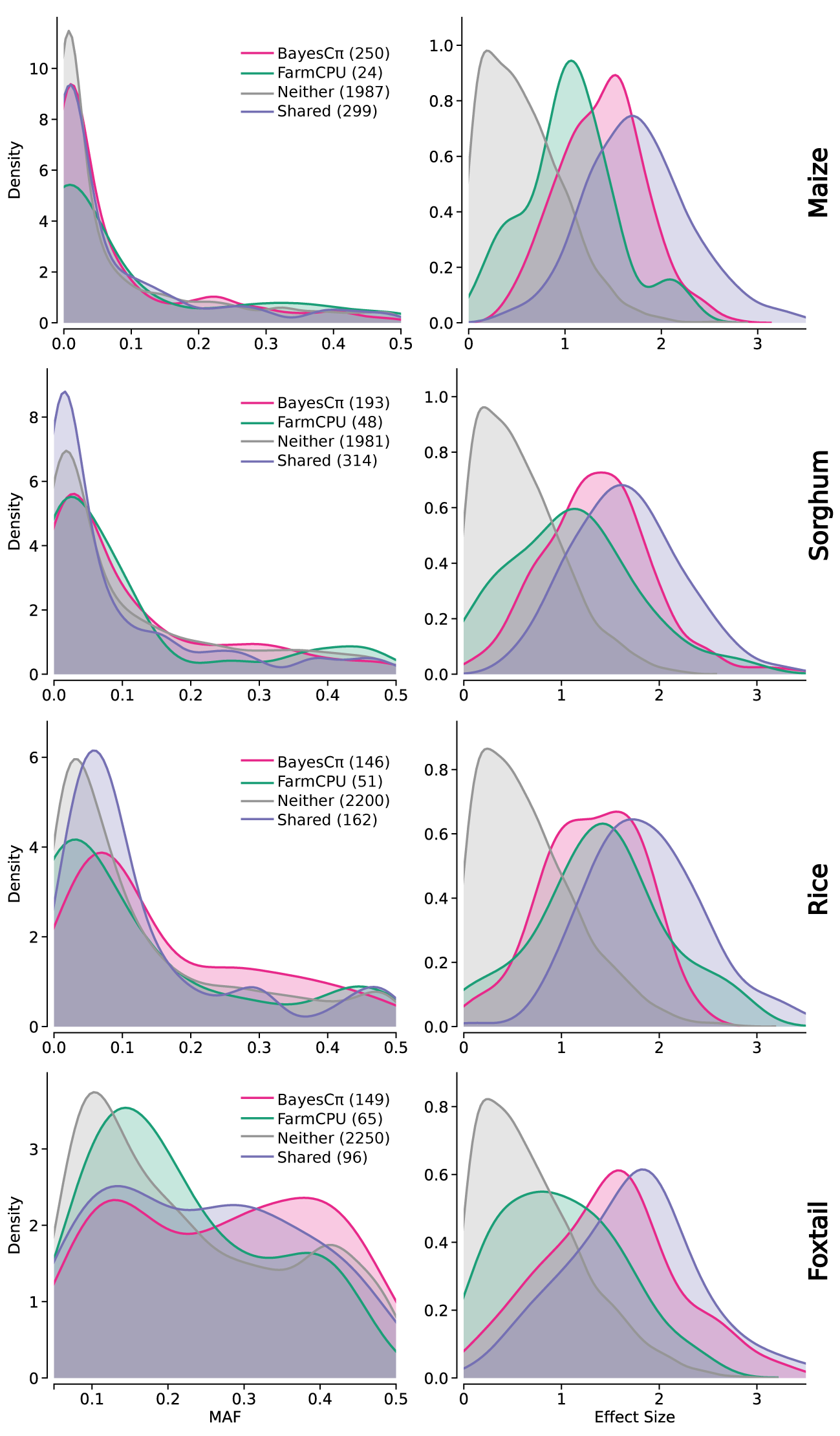


**Figure S15**. Differences in the characteristics of causal SNPs identified by BayesCπ and FarmCPU. Distribution of MAF (Left) and absolute effect size (Right) for causal variants identified by both BayesCπ and FarmCPU, only BayesCπ, only FarmCPU, or neither approach. The number of causal variants in each category is indicated as part of the legend of each panel. Data shown are collected from 10 replicates with 256 causal variants and 0.7 heritability in each species.


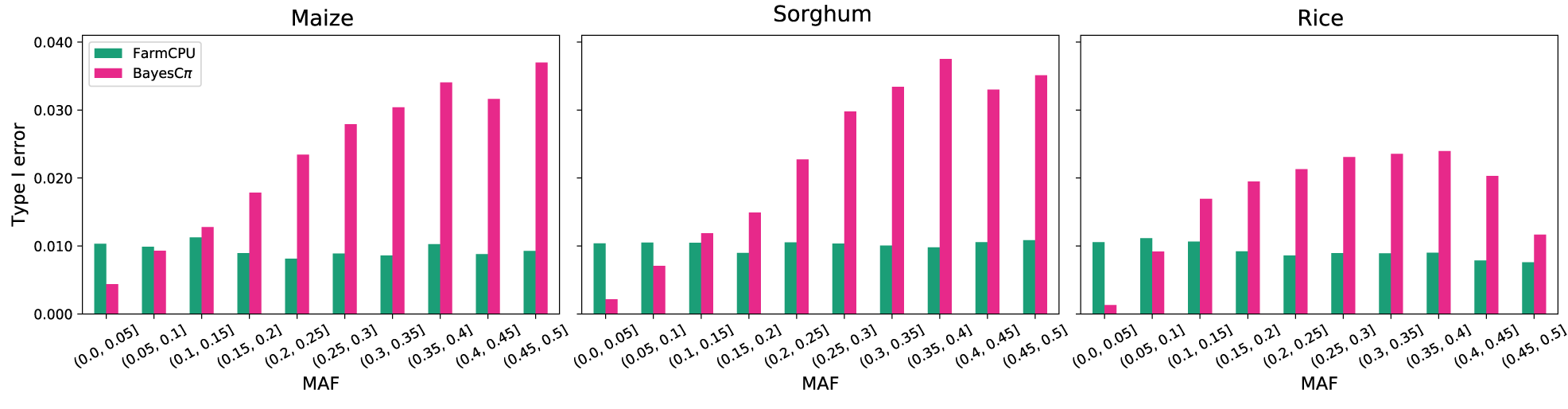


**Figure S16**. Relationship between minor allele frequency and type I error rates for markers in the maize, sorghum, and rice datasets for both FarmCPU and BayesCπ. The foxtail millet dataset lacked rare SNPs and is not shown.


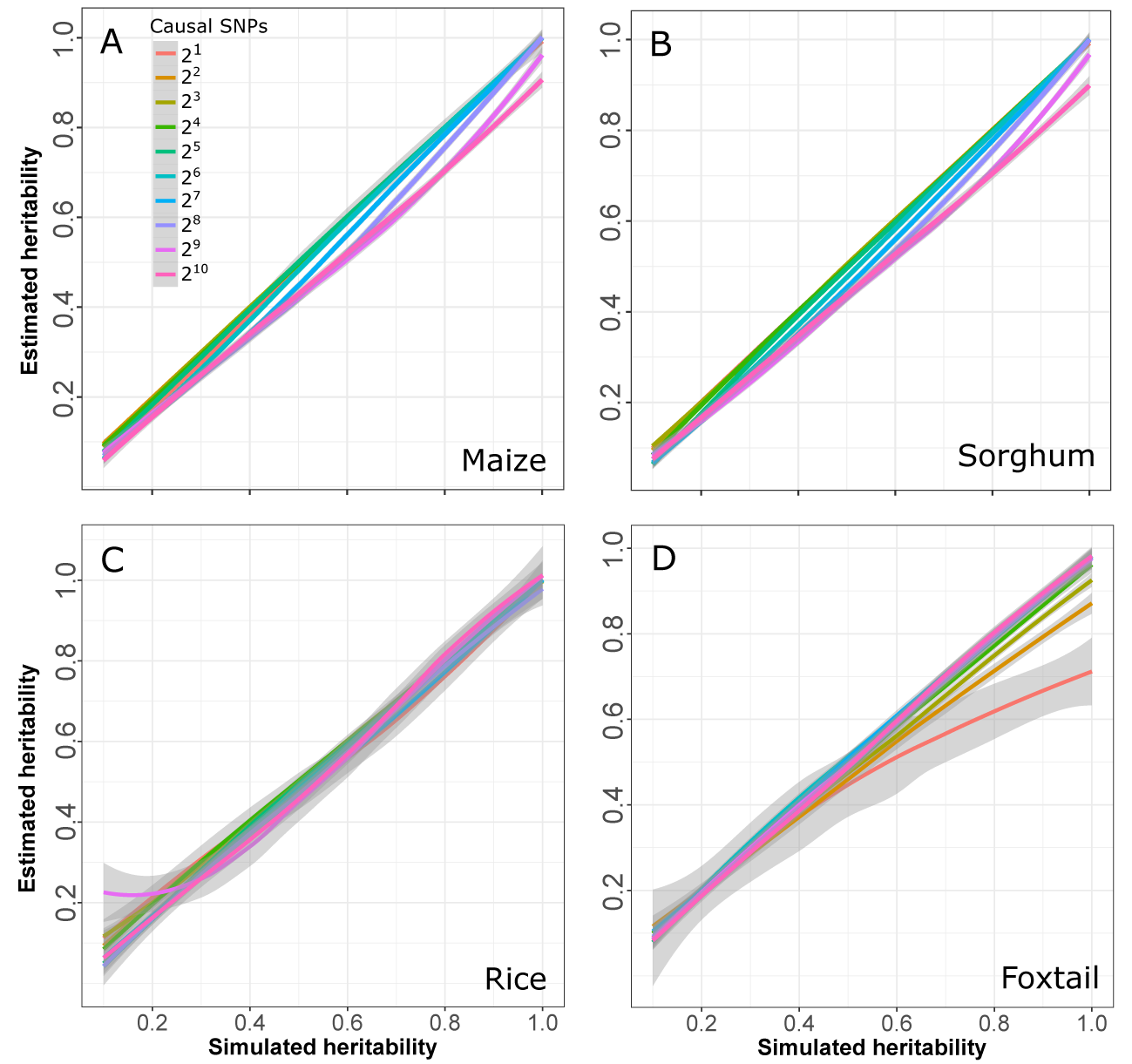


**Figure S17**. Relationship between simulated heritability and heritability estimates generated by BayesCπ for traits controlled by different numbers of causal variants. Grey lines indicate the 95% confidence intervals.


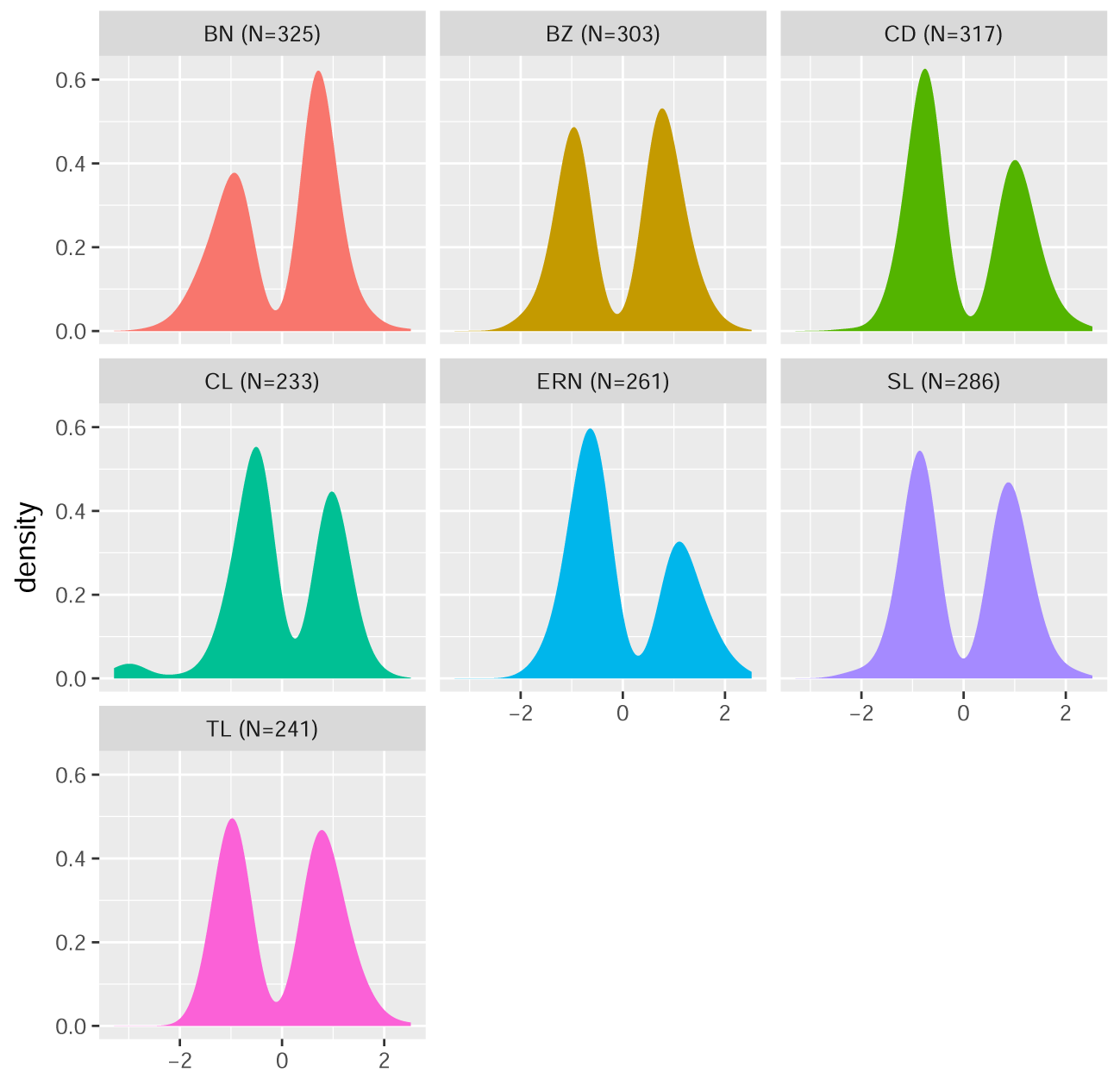


**Figure S18**. Empirically determined effect sizes for loci control seven different traits in maize. These are standardized scaled SNP effects from the GWAS results obtained from (Brown *et al*., 2011). In each case effects were normalized to a mean of 0 and a standard deviation of 1. In most cases, the distribution of effect sizes approximates the two tails of a normal distribution, with a missing center of unidentified small effect value SNPs. BN, branch number; BZ, branch zone; CD, cob diameter; CL, cob length; ERN, ear row number; SL, spike length; TL tassel length.


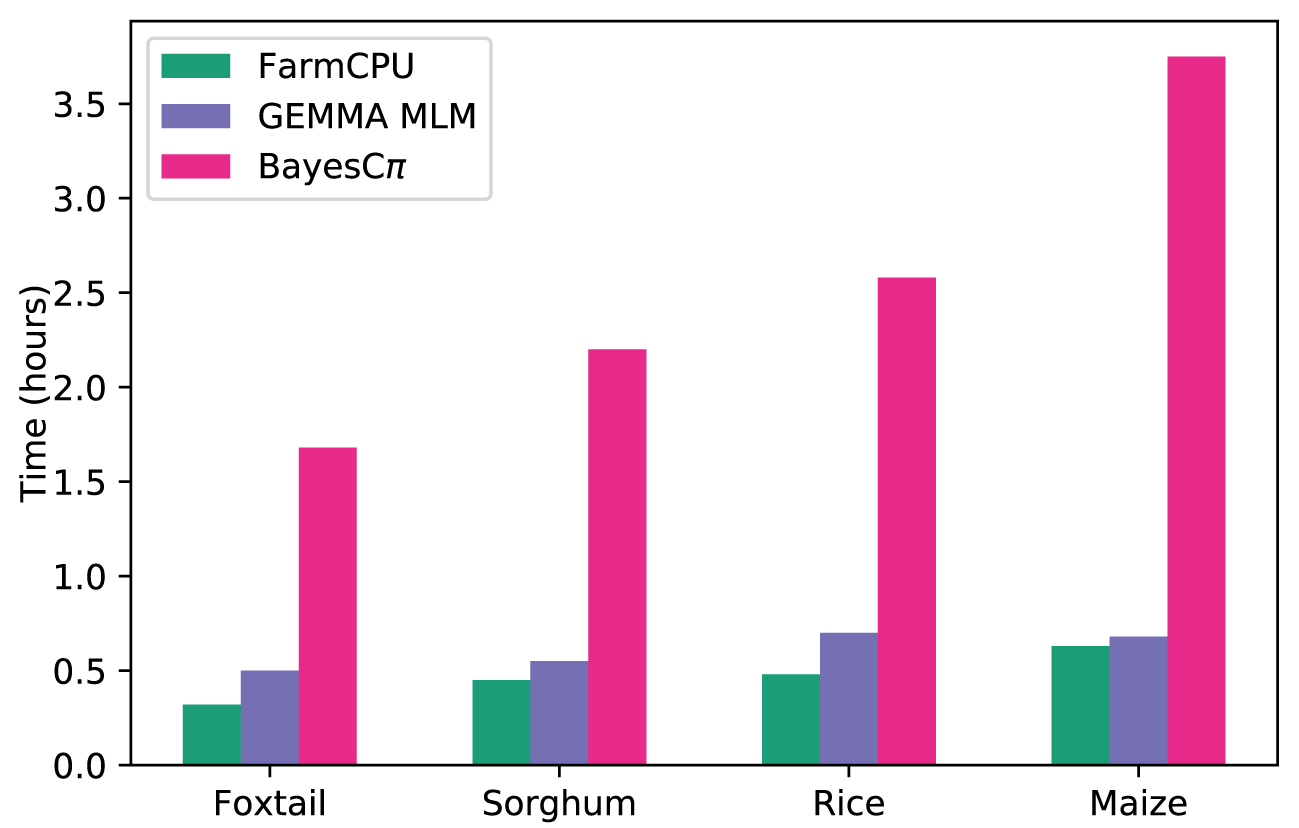


**Figure S19**. Average run time of a single GWAS analysis using each of the three methods evaluation in each of the four populations tested.


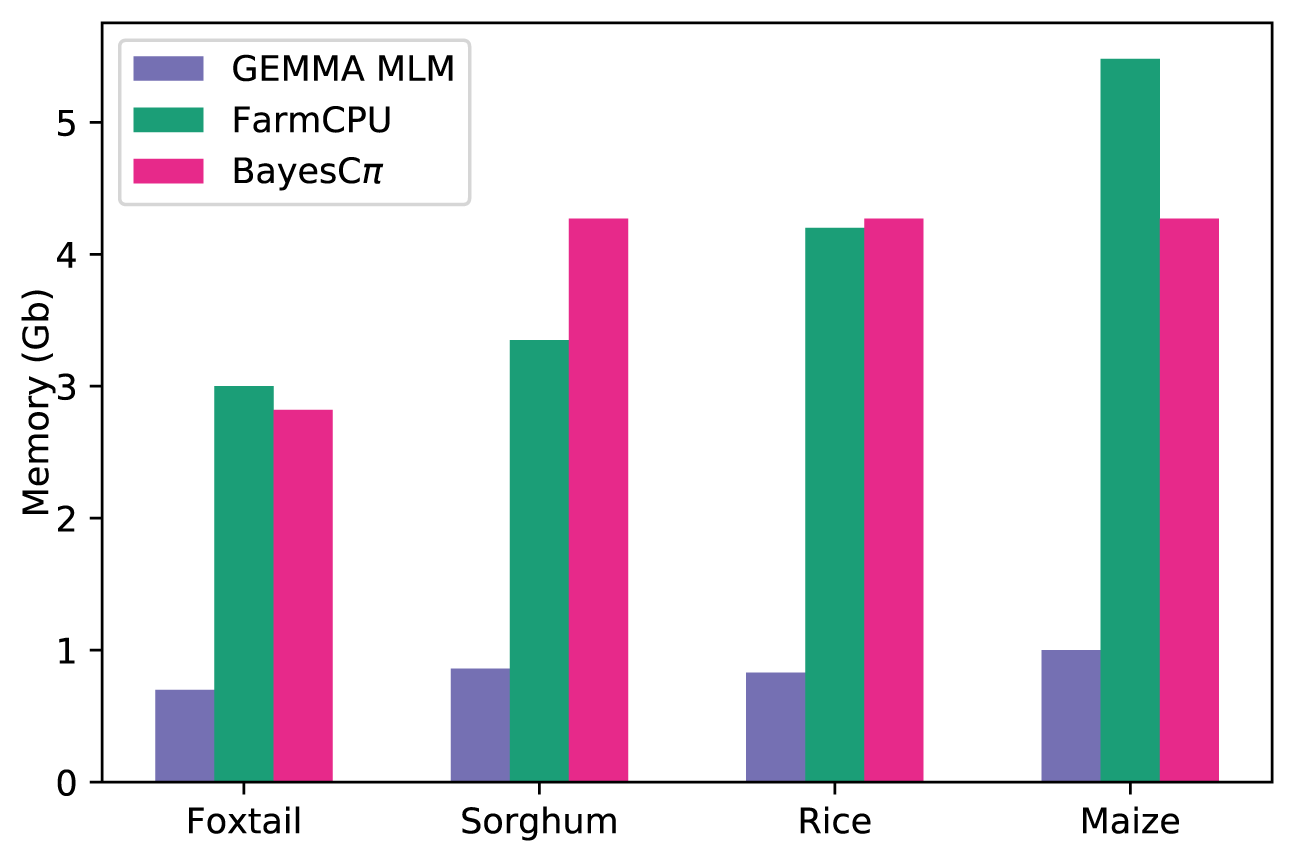


**Figure S20**. Average maximum memory use of a single GWAS analysis using each of the three methods evaluation in each of the four populations tested.


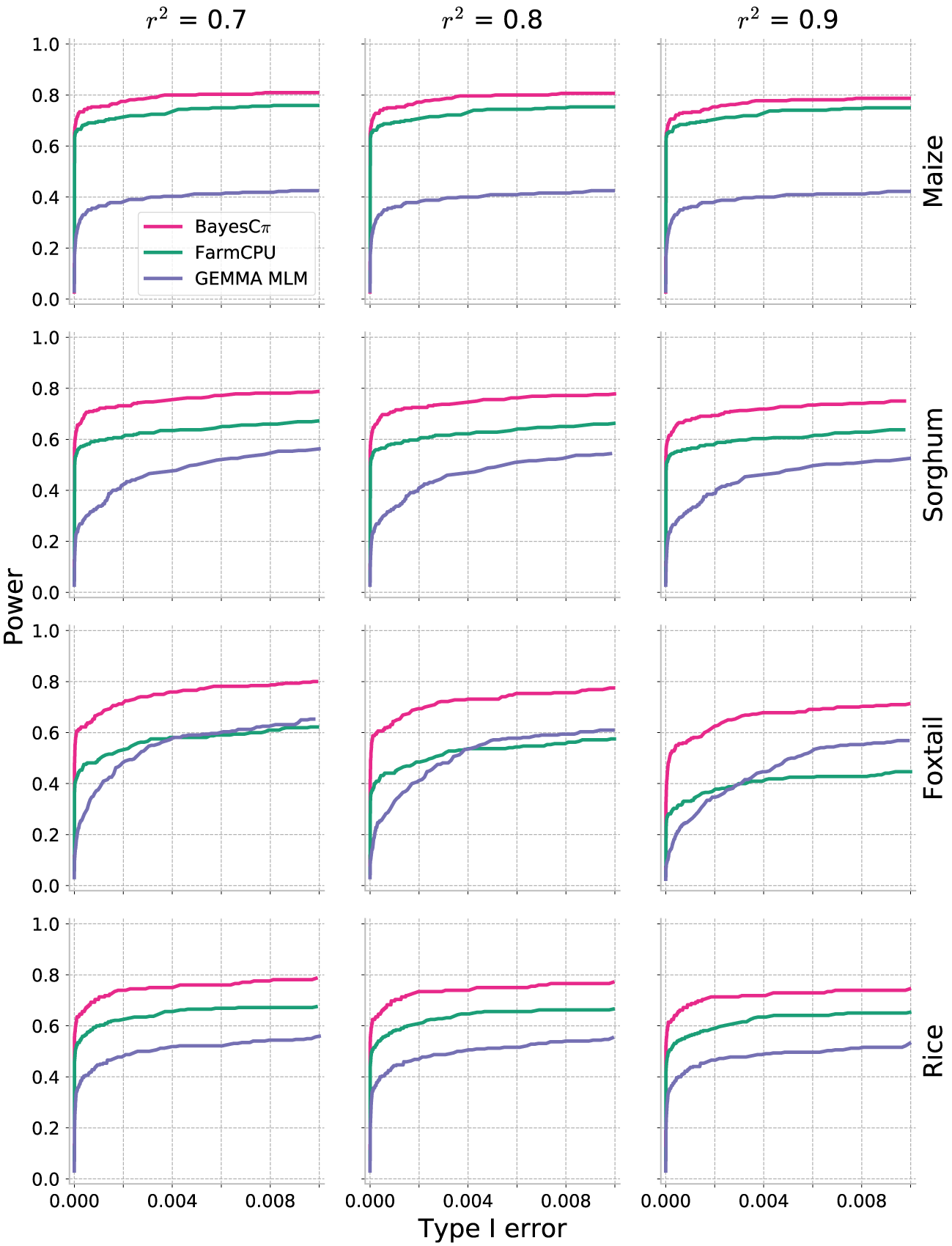


**Figure S21**. The influence of different LD decay cutoffs on apparent GWAS power. Data shown are from simulations where the number of causal variants is 32 and heritability is 0.7.
