# Supplementary material for: Optimising the identification of causal variants across varying genetic architectures in crops
Source: Plant Biotechnol J. 2018 Nov 9;17(5):893–905. doi: 10.1111/pbi.13023 (PMC6587547; doi:10.1111/pbi.13023)
Supplement: Supplementary file 2 — Table S1. Mann–Whitney U test between SNP groups detected and undetected by GEMMA. Data shown are from simulations where the number of causal variants is 64 and heritability is 0.7. Table S2. Mann–Whitney U test between SNP groups identified by FarmCPU, BayesCπ, both and neither. Number of causal SNPs is 256 and heritability is 0.5. [file PBI-17-893-s003.docx]

**Table S1.** Mann-Whitney U test between SNP groups detected and undetected by GEMMA

| Species | Minor allele frequency | Effect size |
| --- | --- | --- |
|  | Detected v.s. Undetected | Detected v.s. Undetected |
| Foxtail millet | 0.33 | 2.88E-27 |
| Sorghum | 0.0029 | 5.5E-42 |
| Maize | 3.77E-14 | 1.06E-45 |
| Rice | 0.089 | 3.35E-44 |

**Table S1.** Mann-Whitney U test between SNP groups detected and undetected by GEMMA. Data shown are from simulations where the number of causal variants is 64 and heritability is 0.7.

**Table S2.** Mann-Whitney U test between SNP groups identified by FarmCPU, BayesCπ, both, and neither

| Species | Minor allele frequency | | | Effect size | | |
| --- | --- | --- | --- | --- | --- | --- |
|  | FarmCPU  v.s.  Shared | BayesCπ  v.s.  Shared | FarmCPU  v.s.  BayesCπ | FarmCPU  v.s.  Shared | BayesCπ  v.s.  Shared | FarmCPU  v.s.  BayesCπ |
| Foxtail millet | 0.0002 | 0.40 | 3.20E-6 | 0.013 | 2.98E-6 | 0.068 |
| Sorghum | 4.09E-17 | 0.0046 | 2.56E-24 | 0.011 | 0.0055 | 0.38 |
| Maize | 2.08E-12 | 0.0039 | 2.39E-19 | 0.0011 | 8.05E-5 | 0.18 |
| Rice | 0.0091 | 0.0005 | 1.08E-5 | 0.00097 | 0.0042 | 0.23 |

**Table S2.** Mann-Whitney U test between SNP groups identified by FarmCPU, BayesCπ, both, and neither. Number of causal SNPs is 256 and heritability is 0.5.
